# Quantum Dot Supraparticle Photocatalysts for Photodegradation of Rhodamine B

**DOI:** 10.1021/acsomega.5c13184

**Published:** 2026-04-10

**Authors:** Charlotte J. Eling, Nicolas Laurand

**Affiliations:** Institute of Photonics, Department of Physics, SUPA, 3527 University of Strathclyde, Glasgow G1 1RD, U.K.

## Abstract

Metal oxide nanomaterials, such as TiO_2_, are extensively utilized in photocatalysis for applications, including water purification, antibacterial disinfection, and energy harvesting. However, the wide bandgap (∼3.2 eV) of TiO_2_ constrains excitation to UV light, limiting its efficiency under solar irradiation. To extend photocatalytic activity into the visible spectrum, colloidal semiconductor quantum dots (QDs) can function either as independent photocatalysts or as sensitizers; in the latter case, facilitating charge transfer to TiO_2_ and enhancing reactive oxygen species (ROS) generation. Here, we demonstrate a photocatalytic platform, composed of QD supraparticles (SP), optionally coated with a titania shell. This hierarchical SP architecture bridges the electronic and photonic scales, significantly enhancing light-harvesting efficiency compared to conventional QDs or metal oxide nanocrystals. The photocatalytic performance of these QD-based SPs is systematically evaluated under both UV and white light illumination, using rhodamine B (RhB) degradation as a model reaction, and compared to QDs and TiO_2_ nanoparticles. We find that SPs facilitate both RhB degradation and *N*-deethylation, with titania-coated SPs (SP/TiO_2_) achieving full transformation to Rhodamine 110. We also show that for QDs and SPs with comparable overall surface area, SPs degrade RhB much faster under both UV and white light irradiation. In addition, the reusability of the QD-based SPs is dramatically improved compared to that of QDs. These findings demonstrate the strong potential of QD-based SPs as photocatalytic materials for environmental, energy, chemical, and biomedical applications.

## Graphical abstract

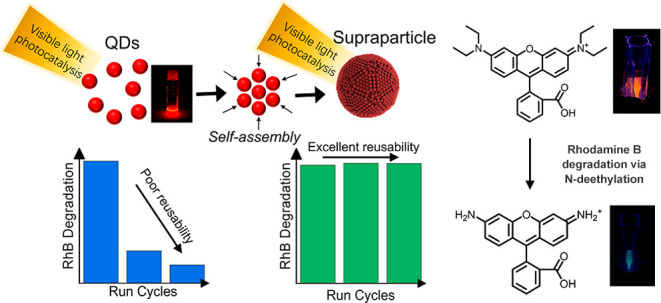

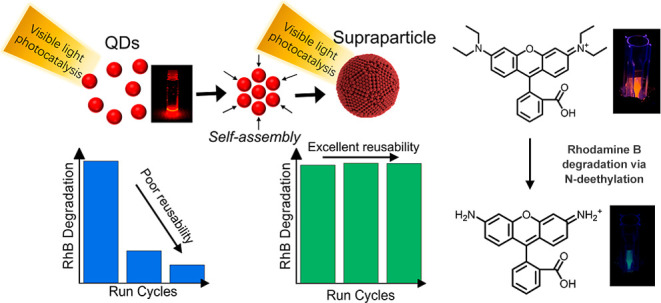

## 1 Introduction

Semiconductor photocatalytic technology has garnered interest over the past decade. The many fields that can benefit from this technology include, but are not limited to, water purification,^1,2^ antibacterial disinfection,^3–5^ energy harvesting, and hydrogen production.^6,7^

Semiconductor photocatalysts work by the photoexcitation of charge carriers. The electron is promoted from the valence band (VB) to the conduction band (CB), leaving a positively charged hole (h^+^) in the VB. The electron and/or hole can migrate to the surface and react with molecules adsorbed onto the material surface. The hole is a strong oxidizing agent that can directly oxidize adsorbed molecules or, when in the presence of molecular oxygen or water, can produce oxygen radicals. The electron is a reducing agent which, when in the presence of water, can reduce it to hydrogen, a clean energy source. It can also reduce other molecules such as CO_2_ and N_2_ into useful byproducts such as CO and ammonia.

To enhance the semiconductors’ photocatalytic ability, we must enhance the absorption of light by the semiconductor and reduce the charge recombination of photogenerated carriers. Take, for example, one of the most researched photocatalysts TiO_2_, with a band gap of 3.2 eV; it can only absorb ultraviolet light of wavelengths 385 nm or less, which accounts for approximately 3–5% of the solar spectrum.^8^ This severely restricts the photocatalytic efficiency of TiO_2_ under solar irradiation. Semiconductors with band gaps of *E*_bg_ < 3 eV can absorb larger amounts of the solar spectrum covering the majority of visible light. This has led to an increase in interest in different semiconductors which absorb in the visible range, in particular, semiconductor quantum dots (QDs). A range of quantum dot architecture has been explored, with efforts focused on heterostructured metal chalcogenide QDs such as core/shell or core/shell/shell QDs, for example, CdSe/CdS/ZnS.^9,10^ These types of QDs have excellent light harvesting abilities in the visible region, which has been shown to improve photocatalytic hydrogen production compared to core-only CdSe QDs.^10^ The spatial separation of excited carriers in the core and shell reduces the probability of electron–hole recombination, which also increases photocatalytic efficiency.

However, chalcogenide QDs are susceptible to photodegradation. This causes irreversible damage and hinders the catalytic activity and use at larger scales. There are two main pathways that lead to degradation in an aqueous environment. First, an accumulation of excess holes results in the formation of elemental sulfur and leached cadmium ions. Second, surface sulfides react with molecular water or oxygen to form sulfates.^11^ A potential solution to reduce photodegradation effects is to use larger, micron-size particles such as supraparticles (SPs).^12–14^ Formed from clusters of smaller particles, and in this case QDs, SPs can offer advantages to their nanoparticle subcomponents while still offering the same and sometimes higher catalytic activity.^12,14^

QD SPs play a distinctive role in photocatalysis by combining collective optical and photophysical effects that are absent in isolated quantum dots.^14,15^ When assembled into SPs with dimensions comparable to the wavelength of light, they function as optical microcavities that support Mie-type resonances.^14,16,17^ These resonances enhance the local electromagnetic field inside and near the SP, leading to increased light absorption by the constituent QDs.^16^ In addition, strong optical resonances prolong the photon dwell time within the SP, increasing the probability of photogenerated charge carriers and therefore photocatalytic efficiency. Beyond purely optical effects, the close packing of quantum dots gives rise to emergent photophysical behavior such as modified exciton dynamics, enhanced energy or charge transfer, and altered recombination pathways, which cannot be achieved with dispersed quantum dots.^16,18^ These collective photophysical effects make QD SPs particularly beneficial architectures for boosting the photocatalytic efficiency.

The photocatalytic activity of SPs composed of mixtures of QDs and Au nanoparticles has been studied;^13^ however, no studies have investigated coating QD-only SPs in metal oxide layers. As the photocatalytic reaction occurs at the material surface, adding a metal oxide outer layer means the reaction will take place on that surface while also promoting charge transfer from QD to metal oxide. As the SPs are made solely of QDs, they provide self-supported mechanical strength for better reusability.^12^ An additional benefit of using SPs is that due to their larger size compared to their subcomponents, the absorption cross-section is much greater, resulting in a greater rate of excited charge carriers. The micrometer-sized SPs are also much easier to separate from the product, compared to nanoscale QDs. In addition, SPs can offer various modalities as a result of the ability to be built from multicomponent building blocks.^14^

Hereon, we present the photocatalytic capability of QD SPs. The SPs are synthesized using bright CdSSe/ZnS alloyed quantum dots with quantum yields of >50%.^19^ To determine their performance as photocatalysts, SPs were tested alongside QDs and TiO_2_ nanoparticles (NPs). In an attempt to further enhance the photocatalytic ability, we report the synthesis and photocatalytic nature of novel constructs of QD SPs coated in a TiO_2_ shell (SP/TiO_2_). This serves several benefits: increases the spectral range of the TiO_2_ to visible light^20,21^ and reduces charge recombination in semiconductor quantum dots by electron transfer from QD to titania.^8,20–23^ QDs such as CdS and CdSe, for example, have been studied as sensitizers for TiO_2_ due to their small bandgap and high CB level.^24–26^ The higher CB level allows for photogenerated electrons in the QDs to be injected into the TiO_2_ CB, separating the oxidation and reduction reaction sites and reducing the chance of charge recombination. Consequently, TiO_2_ can generate reactive oxygen species (ROS) under visible light, significantly improving photocatalytic efficiency.^21,25,27^

Beyond photocatalysis, QD SPs exhibit additional properties, as noted previously. For example, they function as optical microresonators.^28–30^ Owing to the high refractive index of the QDs, Mie resonances can form within the SPs, enhancing absorption and emission compared to individual QDs. Due to the sensitivity of Mie resonances to the local refractive index, SPs can also act as sensors.^31^ Similar sensors have been used to detect single virus particles,^32^ for in vivo sensing,^33–35^ and for monitoring the contractility of cardiac tissue.^36^ Therefore, QD SPs can be employed as multifunctional materials that are capable of detecting and destroying nearby organic materials or bacteria. This could be significant for applications in defense, medicine, and biosensing.

To assess the photocatalytic performance, we monitored the degradation of an organic fluorophore under both UV and visible light. This comparison allowed us to determine whether individual QDs or QD SPs exhibit superior activity. For reference, their performance was benchmarked against TiO_2_, the most widely used photocatalyst.

## 2 Experimental Section

### 2.1 Materials

Chloroform and ethanol were supplied from VWR. Tetraethyl orthosilicate (TEOS), poly­(vinyl alcohol) (PVA), polyvinylpyrrolidone (PVP), ammonia (2 M in ethanol), titanium butoxide, and TiO_2_ NPs (Anatase, <25 nm) were supplied from Sigma-Aldrich. CdS_*x*_Se_1–*x*_/ZnS QDs were purchased from CD Bioparticles (DNP-C006).

### 2.2 Supraparticle Synthesis

CdSSe/ZnS QDs with an excitonic absorption wavelength of 610 nm and peak emission of 621 nm were used as the building blocks to form the SPs using an oil-in-water emulsion technique. The QDs were supplied in toluene with oleic acid ligands. The QDs were precipitated with methanol and then resuspended in 100 μL of chloroform at a concentration of 20 mg/mL. A 1.25% w/w solution of PVA in 450 μL of Milli-Q water was vigorously mixed with the QD solution using a vortex mixer at 2000 rpm for 5 min to create an emulsion. The solution was left to stir at room temperature for 2–6 h to allow all the chloroform to evaporate. The mixture was then centrifuged at 10,000 rpm for 10 min, and the pellet was resuspended in water. The size of the supraparticles were measured with electron and optical microscopy and found to have an average diameter of 1.5 μm with a standard deviation of 1.1 μm. Scanning electron microscopy (SEM) and energy dispersive X-ray spectroscopy were acquired using a JEOL JSM-IT100 InTouchScope with a built-in EDX detector.

### 2.3 Silica Shell Growth

The supraparticles were resuspended in 250 μL of ethanol to form a 2 mg/mL solution. Polyvinylpyrrolidone (PVP) was dissolved in milli-Q water to form a 50 mg/mL solution. In a microcentrifuge tube, 80 μL of SP solution was mixed with 66 μL of PVP solution and sonicated for 20 min. A further 80 μL of SP solution and 66 μL of PVP solution were added and again sonicated for 20 min; this was repeated one further time. The mixture was centrifuged at 10,000 rpm for 10 min and resuspended in ethanol; this was repeated a further 2 times to ensure the removal of unbound PVP. Tetraethyl orthosilicate (TEOS) was mixed with ethanol to form a 635 mM solution. The solution of PVP-SP was mixed with 20 μL of the TEOS solution and sonicated for 5 min. After sonication, 800 μL of DI water and 800 μL of ammonia were added and left to sonicate for 1 h. The solution was centrifuged at 10,000 rpm for 10 min, and the pellet was redispersed in water. This step was repeated 2 more times for purification, resulting in SP coated with a silica shell (SP/SiO_2_).

### 2.4 Titania Shell Growth

SP/SiO_2_ was resuspended in 134 μL ethanol to form a 1.8 mg/mL solution; to this, 1.07 μL of an aqueous 0.1 M Tween 20 solution was added. A second solution of 1.34 μL of titanium butoxide was diluted in 134 μL of ethanol. The two solutions were mixed using a vortex mixer and then sonicated for 20 min. After sonication, the mixture was vortexed for 2 h at 7000 rpm to allow the reaction to go to completion. The resultant was centrifuged at 5000 rpm for 2 min then resuspended in ethanol. This was repeated a further 2 times to aid the removal of bare titania nanoparticles.

### 2.5 Phase Transfer of QDs

Oleic acid capped QDs (2 mg/mL) were dispersed in 0.6 mL of chloroform. A 0.6 mL aqueous 0.2 M thioglycolic acid (TGA) solution was made and pH adjusted to 10.2 with a tetramethylammonium solution. The two solutions were mixed together and left to stir vigorously at room temperature for 2 h, after which the QDs resided in the water layer. The QDs were precipitated from the water layer with isopropanol and washed in milli-Q water to remove any unbound ligands. This was repeated a further 2 times and finally suspended in water, where the pH was lowered to 7 using a 0.1 M HCl solution.

### 2.6 Photocatalysis Measurements

The photocatalysis measurements were obtained by mixing a 20 μM RhB solution in water with one of the four photocatalysts, all at a concentration of 1.2 mg/mL: QD (TGA-coated QDs synthesized in 2.5), SP, SP/TiO_2_, or TiO_2_ NPs in a glass sample tube. The mixture was stirred and left to equilibrate in the dark for 30 min. The sample was then irradiated with a UV (Cole-Parmer, 9815-series, 6 W at 940 μW/cm^2^) or white light lamp (Quartz Tungsten-Halogen lamp, Thorlabs, 50 mW at 2.1 mW/cm^2^); the sample was placed 5 cm below the lamp and continuously stirred with a magnetic stirrer. To obtain the absorption spectra of the RhB, 100 μL of the photocatalyst/RhB solution was removed from the sample and centrifuged at 13,300 rpm for 5 min to separate the photocatalyst and supernatant containing RhB. Absorption spectra of the RhB supernatant were taken using a spectrophotometer (Thermo Fisher Genesys 30). The photocatalytic degradation was found by measuring the maximum absorbance of RhB.

## 3 Results and Discussion

### 3.1 Synthesis of Materials

#### 3.1.1 Synthesis of SP

The SPs are synthesized using an oil-in-water emulsion technique (Figure 1A). The QDs were 6 ± 0.5 nm in diameter, as shown by the TEM image in Figure 2A. CdSSe/ZnS QDs were used with an emission peak centered at 620 nm and the first excitonic absorption peak at 610 nm (Figure 2D). The QDs were suspended in chloroform at a 20 mg/mL concentration to form the organic phase, with the water phase consisting of 1.25% w/w poly­(vinyl alcohol). The two phases were mixed to form an emulsion and stirred at room temperature to allow the chloroform to evaporate, resulting in SPs made of densely packed colloidal quantum dots. Scanning electron microscopy (SEM) images (Figure 2B) show SPs are spherical with a relatively smooth surface. The SPs have an average diameter of 1.5 μm with a standard deviation of 1.1 μm, measured via SEM (Figure S1). Elemental analysis of the SPs was performed using energy-dispersive X-ray (EDX) spectroscopy via the SEM. Figure S2 is EDX maps where Cd, Zn, and S were detected, indicating the quantum dots are present in the SPs. The emission peak resides at 624 nm, and the excitonic peak is still visible in the absorption spectra; however, due to the larger size of the SP, more scattering is evident at shorter wavelengths (Figure 2E).

**Figure 1:**
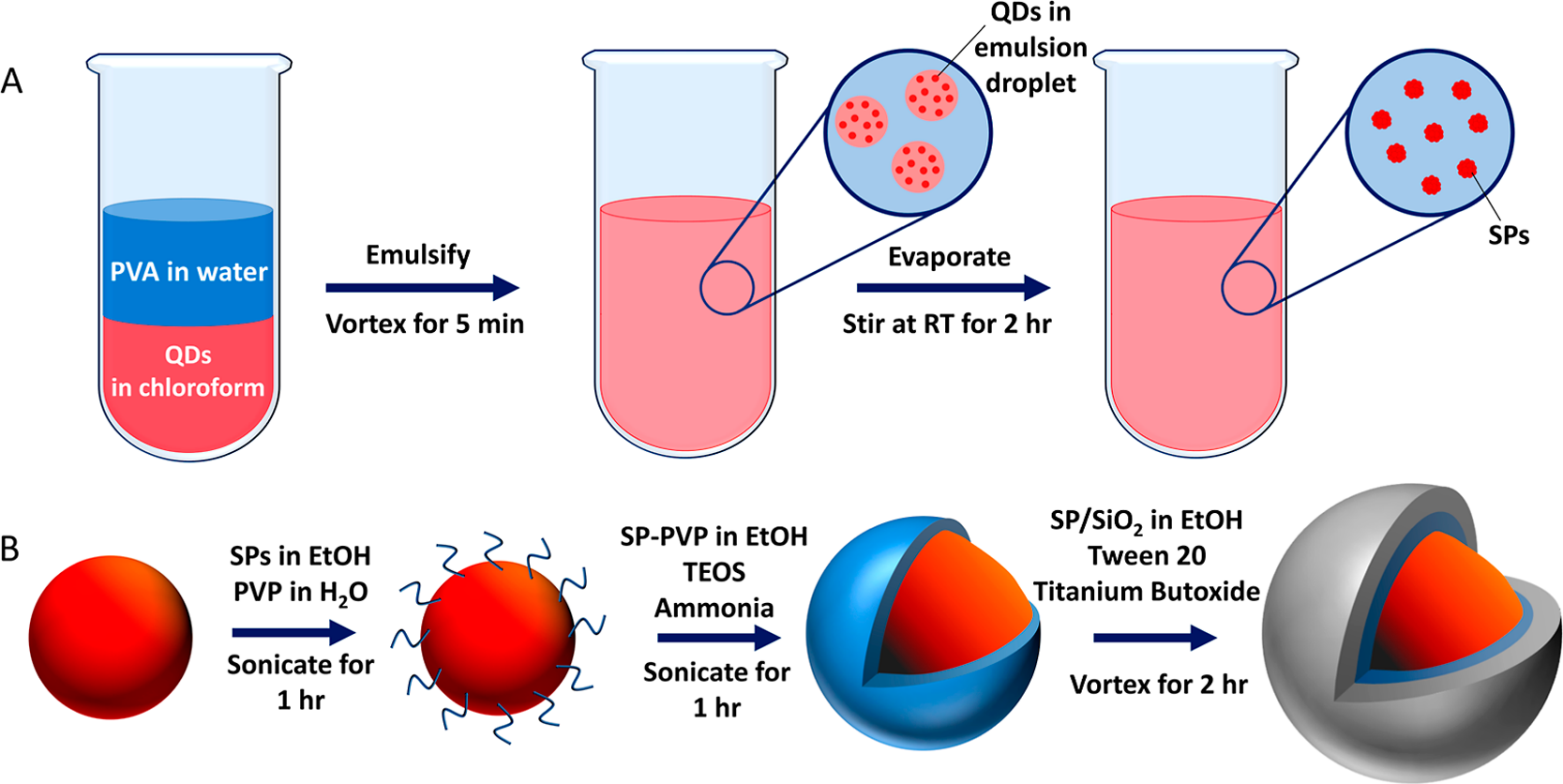
Schematic of supraparticle synthesis. (A) Emulsion of poly­(vinyl alcohol) (PVA) in water with QDs in chloroform was made by vortexing the two solutions together. The chloroform emulsion droplets were left to evaporate, resulting in densely packed QD SPs. (B) SPs were coated in a titania shell via a 3-step process. First, the SPs were functionalized with PVP, followed by deposition of a thin silica layer and then finally titania.

**Figure 2:**
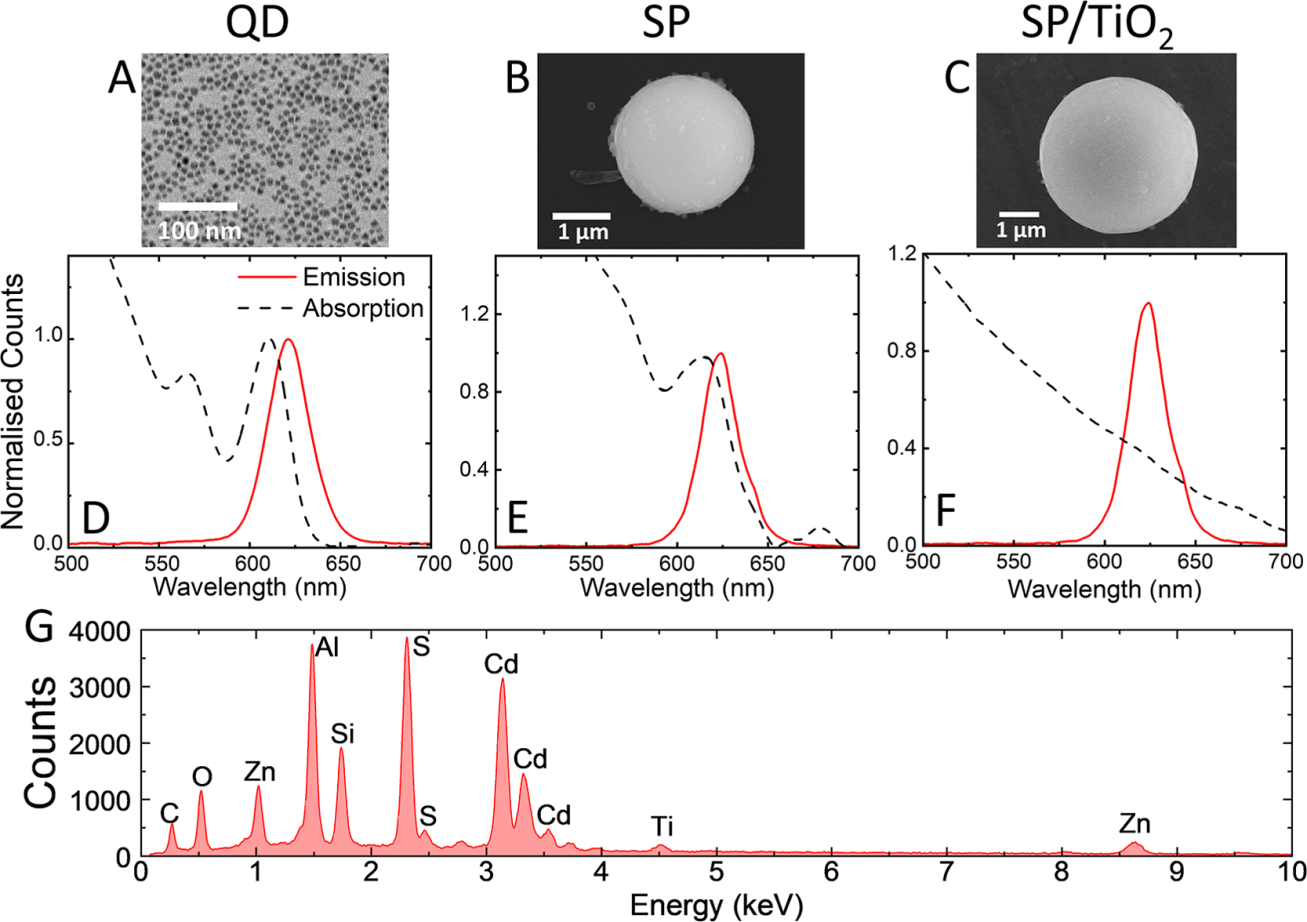
(A) Transmission electron microscope image of QDs. (B,C) Scanning electron microscope (SEM) image of SP and SP/TiO_2_. (D–F) Emission and absorption spectra of QD, SP, and SP/TiO_2_, respectively. (G) Energy dispersive X-ray spectroscopy of SP/TiO_2_ SPs.

#### 3.1.2 Synthesis of SP/TiO_2_

The titania shell was grown onto the surface of the SPs in a two-step process (Figure 1B). First, to allow for water solubility, a thin silica layer (<10 nm) was grown on the surface of the SPs. This was achieved using tetraethyl orthosilicate (TEOS) as the silica precursor and ammonia as the reducing agent, a technique we have previously reported.^30,37^ EDX maps (Figure S3) of the silica-coated SP show silicon and oxygen overlapping the cadmium from the QDs, indicating that there is a coating of SiO_2_ on the SPs. The titania shell was then grown directly on top using a sol–gel method with titanium butoxide and the surfactant tween 20.^28,38^ SP/TiO_2_ had an average diameter of 3.6 μm with a standard deviation of 2.1 μm measured via SEM (Figure S1). SEM images of SP/TiO_2_ (Figure 2C) show that the SP retains its spherical shape after the coating. The presence of the silica and titania shell was confirmed through EDX spectroscopy. Figure 2G shows the EDX spectra of the silica- and titania-coated SP. The peaks at 4.51 and 1.74 keV are representative of titanium and silicon, respectively, indicating the presence of titania on the surface of the supraparticle. The peaks at 3.14, 2.31, and 1.02 keV are representative of cadmium, sulfur, and zinc, respectively, which make up the QDs. EDX maps of SP/TiO_2_ (Figure S4) show the presence of cadmium and zinc from the QDs with titanium and oxygen from the TiO_2_ shell. The two maps of cadmium and titanium were laid over one another, and the evident match shows that the coating was successful. The average increase in SP/TiO_2_ size compared to SP is thought to result from the fragmentation of the smaller SPs (<1 μm) being broken down during the shelling process. After shell growth, the SP/TiO_2_ emission peak remained at 623 nm (Figure 2F). The absorption spectrum shows an exponential trend characteristic of the scattering due to the titania shell. The scattering from the titania is the dominant feature in the extinction spectra.

ζ-Potential measurements of the uncoated SPs, silica-coated (SP/SiO_2_) and silica–titania-coated (SP/TiO_2_) were obtained in water at pH 6.8. The ζ-potentials were −12.2 ± 6.86, −40.3 ± 6.72, and −21.6 ± 17.9 mV, respectively (Figures S5–S7). The reduction in ζ-potential from −12 to −40 mV is due to the presence of deprotonated silanol groups, which increases to −21 mV with the growth of titania. This agrees with values found in literature.^30,39,40^

### 3.2 Samples for Photocatalysis

The performance of four photocatalysts was tested: SP, SP/TiO_2_, TiO_2_, and QDs. The QDs are coated in oleate ligands and therefore are not soluble in water; to ensure the solvent is kept consistent between samples, a phase transfer was performed, exchanging the oleate molecules with TGA to allow for water solubility. After ligand exchange, the ζ-potential of QD-TGA was −31.5 mV ± 5.7 mV (Figure S8). The ζ-potential of the TiO_2_ NPs was found to be −62.3 mV ± 19.7 mV (Figure S9).

### 3.3 Photocatalysis Results

The photocatalysis of QD, SP, and SP/TiO_2_ was tested under UV and white light and compared to a sample of TiO_2_ NPs. TiO_2_ is one of the most known photocatalysts and is often used as a standard to compare new photocatalytic materials. To measure the photocatalyst efficiency, the concentration of each photocatalyst was kept at 1.2 mg/mL. A uniform mass concentration was chosen for simplicity and to reflect realistic operating conditions in practical photocatalytic applications. The photocatalysts were dispersed in a 20 μM aqueous solution of RhB. To negate any photobleaching effects, a control measurement of RhB with no photocatalyst was also irradiated and measured.

After the photocatalysts were mixed with the RhB solution, they were kept in the dark under continuous stirring to equilibrate for 30 min. This was to allow for the adsorption of RhB to the surface of the photocatalysts. After 30 min the light was turned on; this is represented by the dotted lines on Figures 4 and 7. After equilibration, the absorbance had dropped for all samples when compared to the RhB control sample. This is due to a separate catalytic reaction, most likely due to intrinsic metal catalysis or chemical decomposition. To ensure the 30 min equilibrium period was sufficient, control measurements were run whereby a 1.2 mg/mL solution of SP was mixed with 20 μM RhB and a separate 20 μM RhB solution was kept in the dark under continuous stirring for 3 h, with the absorbance measured every 30 min. For the sample with SP, an initial drop in the absorbance down to 76% of its initial value was measured, which then stabilized (Figure S10). Although this causes a decrease in the initial absorption of the dye it is considerably less efficient than the photocatalytic reaction.^1^ The solution of RhB only was much more stable over the 3 h period (Figure S10).

**Figure 3:**
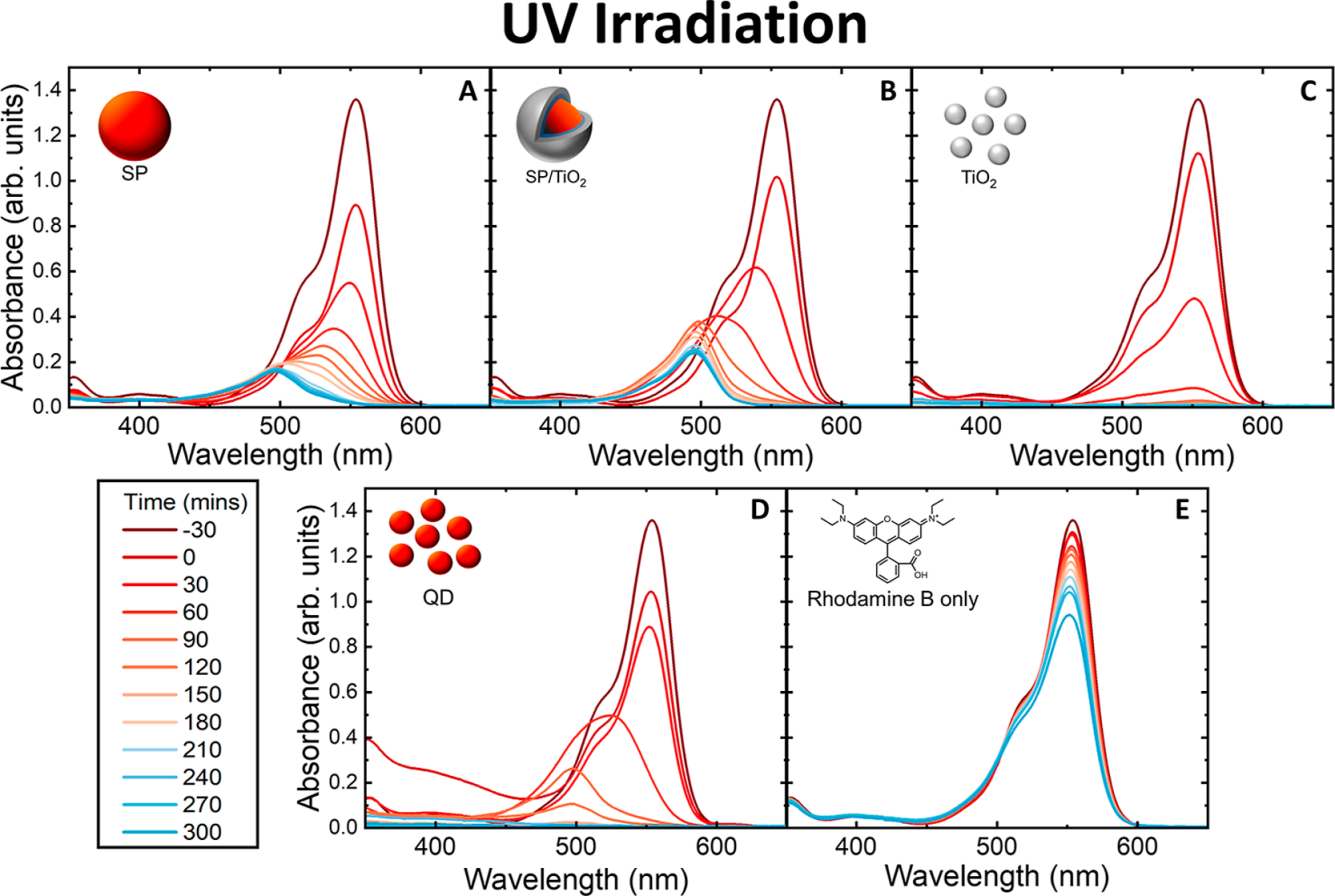
(A–E): Absorbance spectrum of RhB taken at 30 min intervals under UV irradiation with the photocatalysts SP, SP/TiO_2_, TiO_2_, QD, and RhB-only control.

**Figure 4:**
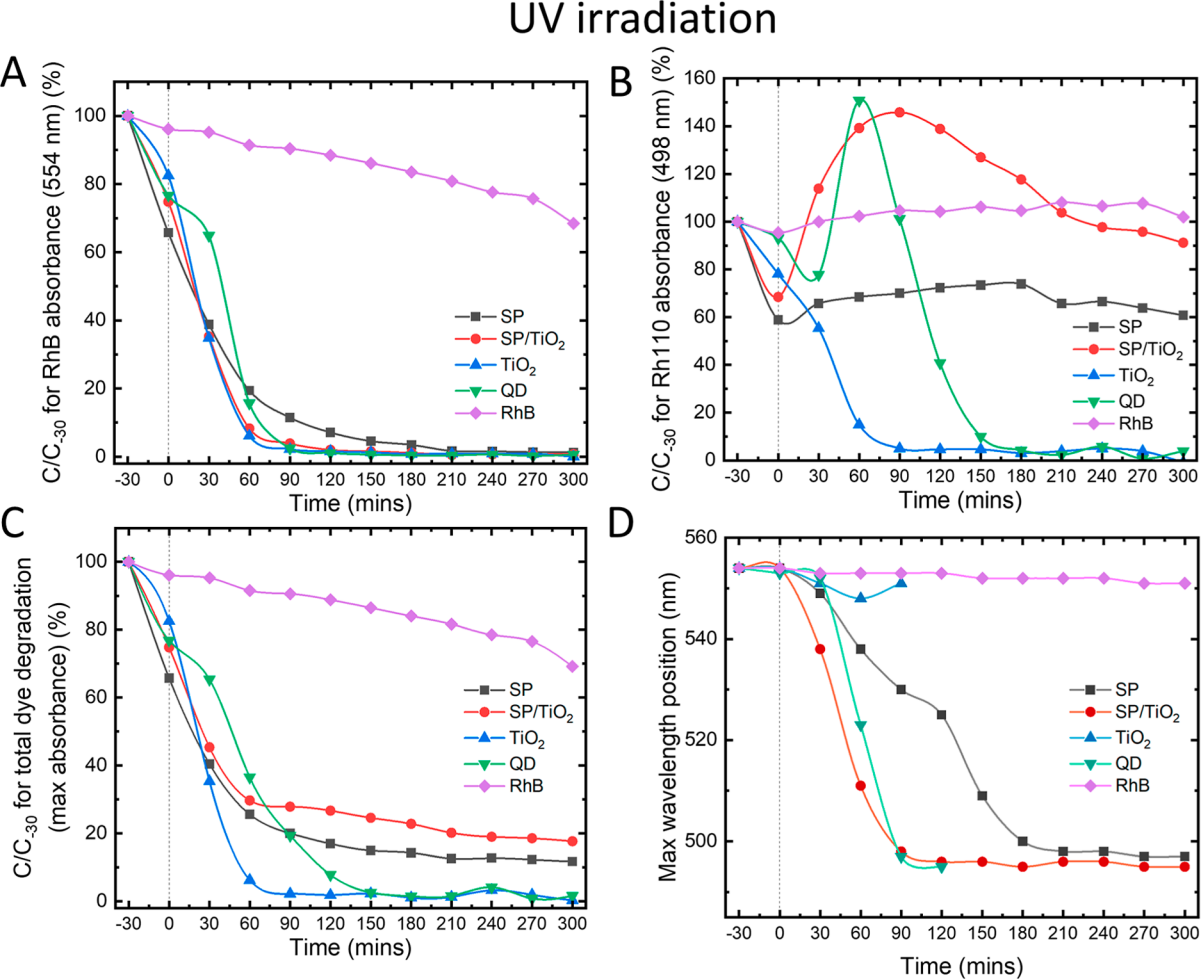
Percentage of photocatalytic degradation of RhB under UV light as a function of time with photocatalysts: SP, SP/TiO_2_, TiO_2_, QD, and RhB-only control. (A) Absorbance monitored at 554 nm, which is the characteristic wavelength of RhB; (B) absorbance at 498 nm, characteristic wavelength of Rh110; (C) maximum absorbance, demonstrating total dye degradation; and (D) maximum absorbance wavelength, representing the transformation of RhB to Rh110. The dotted line at *t* = 0 represents when light is turned on.

**Figure 5:**
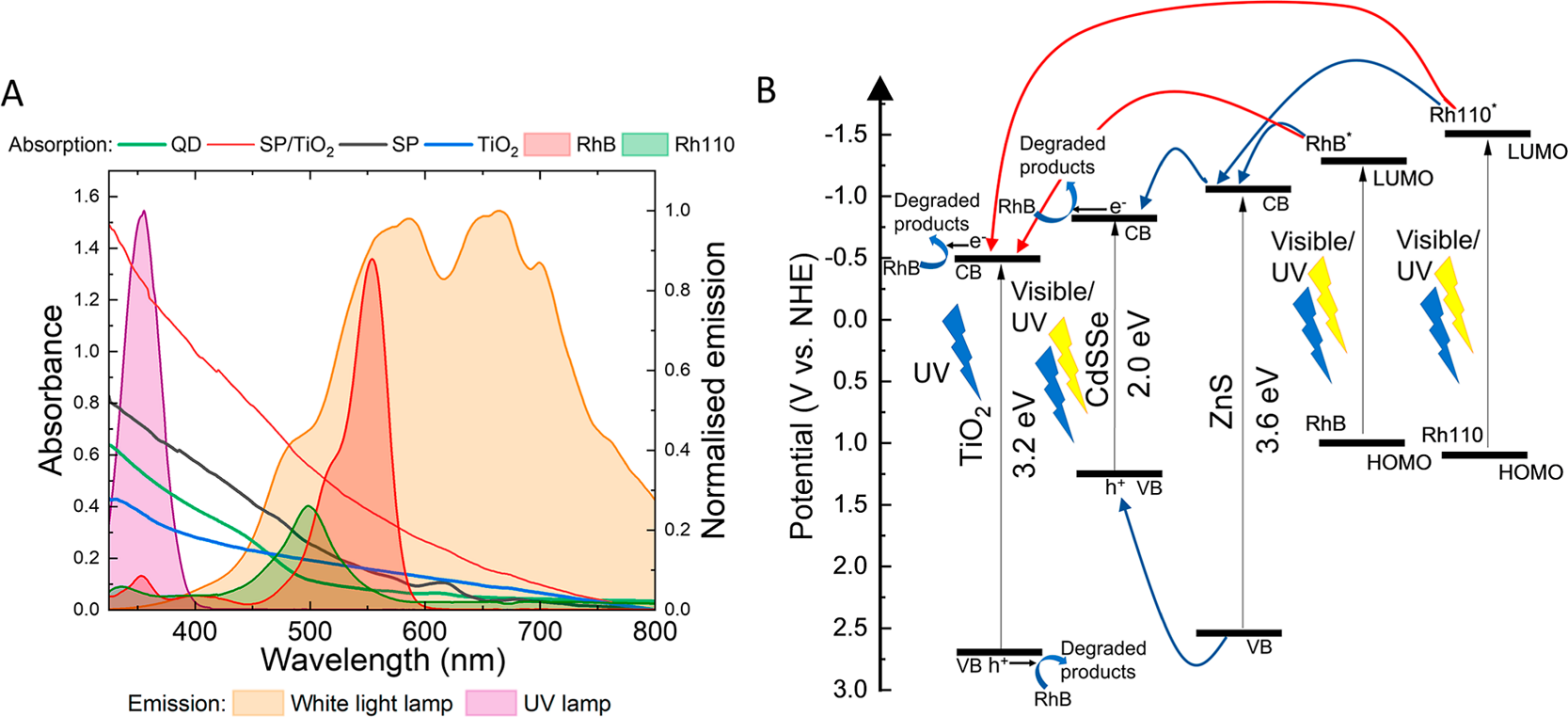
(A) Absorbance spectra of photocatalysts: QD, SP/TiO_2_, SP, and TiO_2_ with RhB and Rh110. The concentration of the photocatalysts was kept the same at 0.3 mg/mL. The concentration of RhB was 20 μM, and the absorption of Rh110 is the fully *N*-deethylated product of RhB. The emission profiles of the UV and white light lamps are also shown. (B) Band-edge alignment with conduction band (CB) and valence band (VB) photoinduced charge-transfer pathways in a Rhodamine-sensitized CdSSe/ZnS and CdSSe–TiO_2_ hybrid system referenced to the NHE (normal hydrogen electrode) scale. The blue arrows indicate electron pathways from excited-state RhB/Rh110 to ZnS and CdSSe cores for QD and SP samples. Red arrows indicate pathways for SP/TiO_2_ and bare TiO_2_ NPs. Values for the band gap of TiO_2_ and ZnS were found from literature.^46^ As the CdSSe QD core is an alloy, an estimate of the band gap was deduced from absorbance and PL spectra taking into consideration the band gaps of CdS and CdSe.^47^ Estimated redox potentials for the rhodamine dyes were found from literature.^44,45^

**Figure 6:**
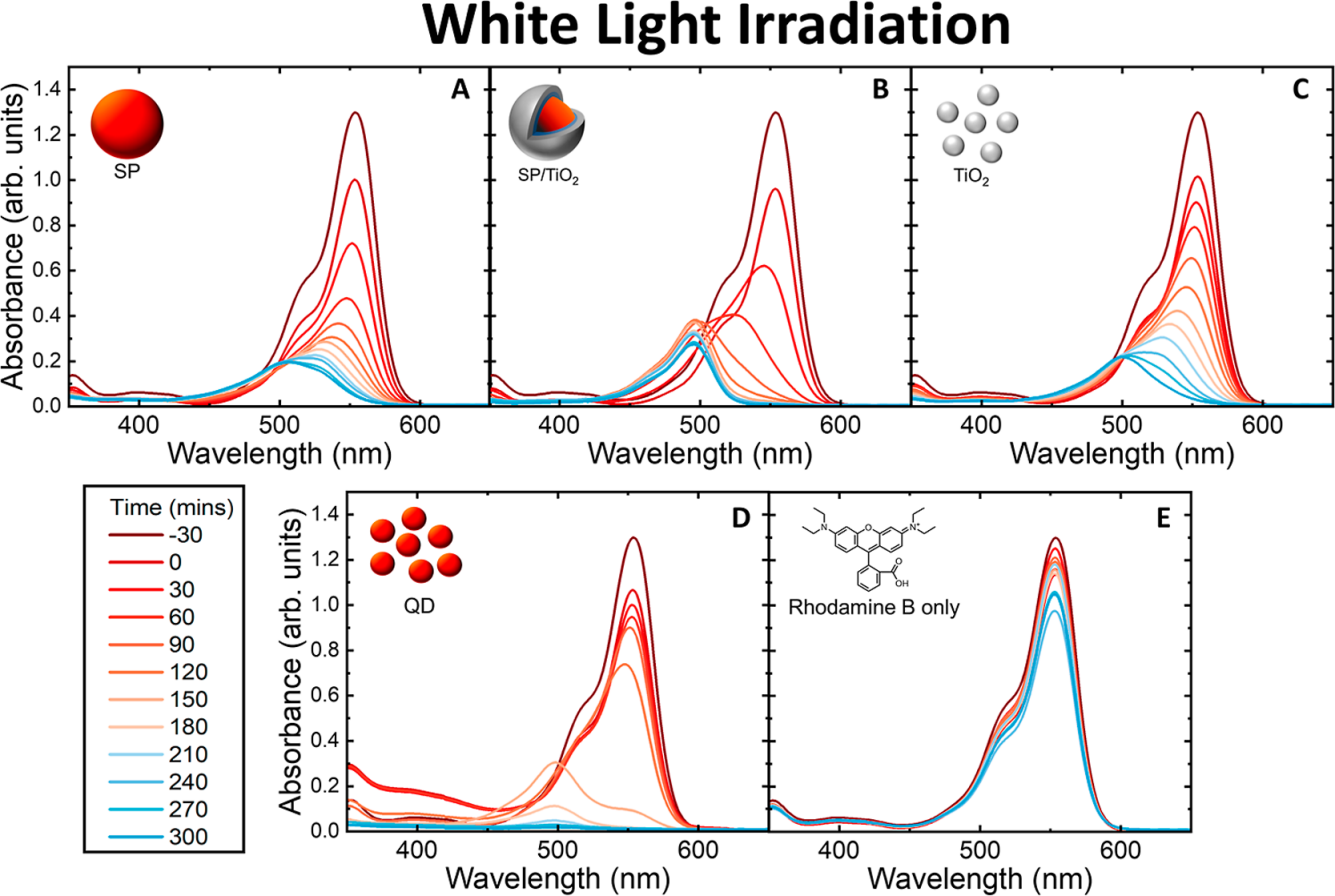
(A–E): Absorbance spectrum of RhB taken at 30 min intervals under white light irradiation with the photocatalysts SP, SP/TiO_2_, TiO_2_, QD and RhB-only control.

**Figure 7:**
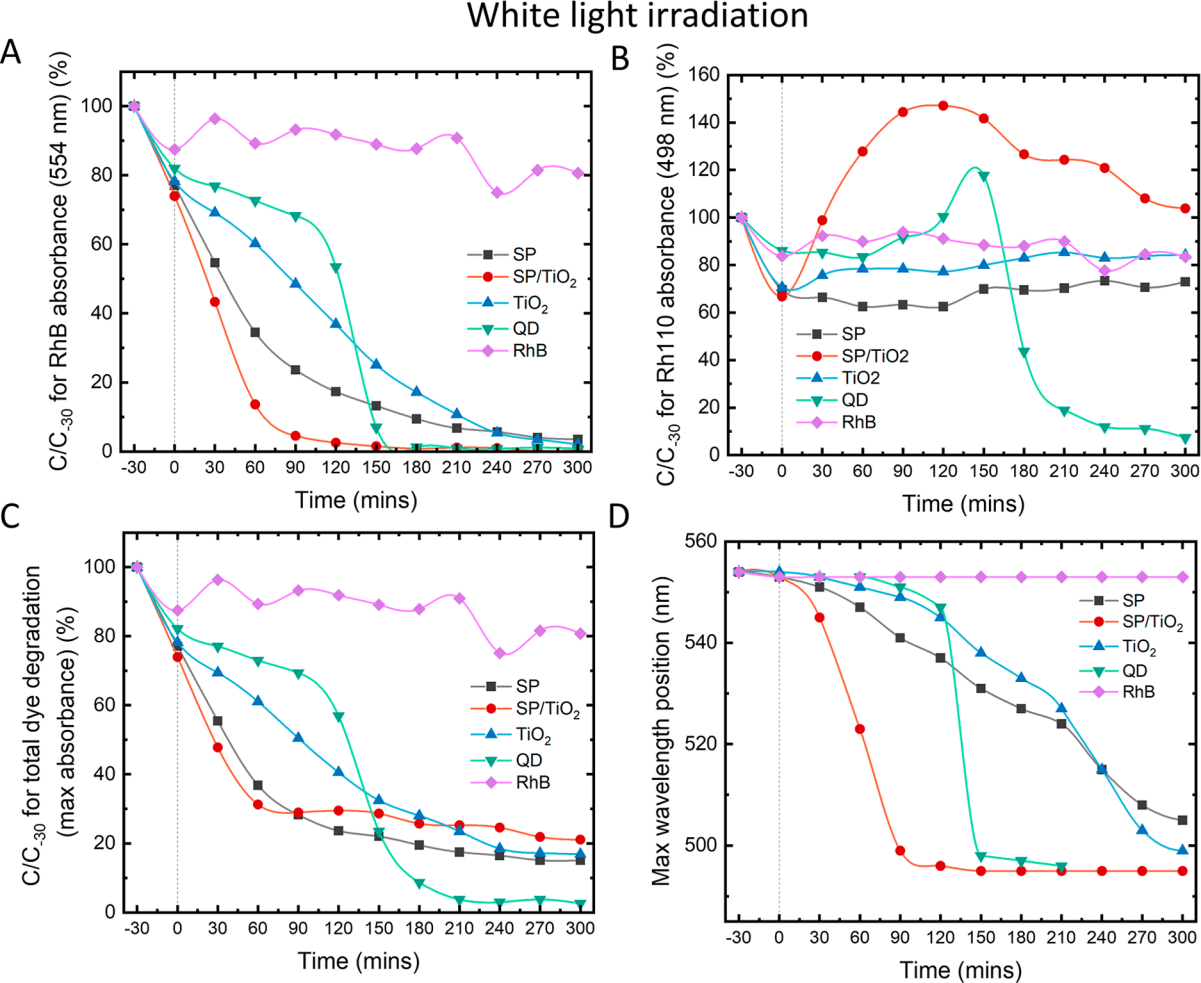
Percentage of photocatalytic degradation of RhB under white light as a function of time with photocatalysts: SP, SP/TiO_2_, TiO_2_, QD, and RhB-only control. (A) Absorbance monitored at 554 nm, which is the characteristic wavelength of RhB (B) absorbance at 498 nm, characteristic wavelength of Rh110 (C) maximum absorbance, demonstrating total dye degradation (D) maximum absorbance wavelength, representing the transformation of RhB to Rh110. Dotted line at *t* = 0 represents when light is turned on.

#### 3.3.1 UV Irradiation

The pathways of RhB degradation with different photocatalysts are evident by the absorption spectra (Figure 3). For TiO_2_, the absorbance of the dye quickly diminishes over a 2 h period due to the cleavage of the chromophore. For photocatalysts SP, SP/TiO_2_, and QD, the degradation of the dye follows a different pattern, most notably with a hypsochromic shift from 554 to 498 nm.

This can be attributed to the *N*-deethylation of RhB, which can occur when the dye is adsorbed onto the surface of the catalyst and is under excitation.^41–43^ This process is due to the RhB molecule donating an electron to the photocatalyst, better known as self-photosensitization. The electron injection from the RhB causes the dye molecule to oxidize to a radical cation, RhB^•+^, resulting in the removal of the –N­(Et)_2_ group. Further excitation of the dye leads to a stepwise removal of the remaining ethyl groups, leading to the completely de-ethylated product, rhodamine 110 (Rh110). The electron injected from the dye molecule into the photocatalyst can then create ROS. Although the indirect generation of ROS and formation of Rh110 are due to the photoinduced electron transfer of dye to photocatalysts, the two are independent processes, therefore can occur simultaneously. *N*-deethylation of RhB occurs from the hydrolysis of the dye cationic radical in the absence of active oxygen species. Therefore, the newly transformed Rh110 can also be fully degraded. In certain applications, Rh110 is preferable to RhB because of its superior biocompatibility, enhanced photoluminescence quantum yield, reduced sensitivity to pH fluctuations, and higher diffusion coefficient.^41^

To understand the degradation in more detail, the percentage of RhB degradation was determined by (*C*/*C*_–30_ × 100), where *C* = concentration of dye at time *t* and *C*_–30_ = concentration of dye when the photocatalyst and dye are first mixed (*t* = −30 min). The samples are mixed in the dark for 30 min, and the light is turned on at *t* = 0.

Figure 4A monitors the degradation of RhB by focusing on its characteristic absorbance maximum at 554 nm. All photocatalysts are able to quickly degrade the dye, with SP/TiO_2_, TiO_2_, and QDs taking only 90 min and SP, 210 min to fully degrade. It must be noted that although the samples are of the same mass concentration, the total surface area varies substantially between the samples, consequently affecting the total number of available reactive sites. Therefore, direct comparison of the times taken to degrade the dye does not reflect the photocatalytic efficiency of the intrinsic material. However, monitoring the absorbance at specific wavelengths gives an insight into the charge transfer pathways of the systems. Figure 4B shows the evolution of Rh110 by monitoring its absorbance at its characteristic absorbance maxima of 498 nm. An increase in concentration of Rh110 indicates the transformation of RhB to Rh110 through *N*-deethylation of the dye. Figure 4C monitors the total dye degradation of RhB, Rh110, and its intermediate derivatives. For TiO_2_, there is little to no transformation to Rh110, with all dye being degraded by just 90 min; therefore, we can deduce that the degradation of RhB is predominantely due to the formation of ROS from the TiO_2_. Figure 5A shows the absorbance spectra of all photocatalysts at the same mass concentration, and Figure 5B is an energy diagram showing band gaps of TiO_2_, the CdSSe core, and ZnS shell quantum dot materials with the estimated redox potentials of RhB and Rh110. Although the absorption of TiO_2_ in the UV irradiation is lower than that of the other photocatalysts (Figure 5A), it is still very effective at degrading the dye. The VB of TiO_2_ is more positive (Figure 5B) compared to the CdSSe and ZnS and therefore has a higher oxidation potential. The photogenerated holes are stronger oxidizing agents compared to those generated in the CdSSe and ZnS, resulting in a higher production of hydroxyl radicals capable of degrading the dye.

The remaining samples SP, SP/TiO_2_, and QD all showed some degree of Rh110 transformation due to the sensitization of the rhodamine dyes. For QD and SP/TiO_2_, there is a sharp increase in the concentration of Rh110 up to 150% (Figure 4B), which then rapidly decreases as the newly formed Rh110 starts to degrade. For SP, although the concentration of Rh110 does not rise above 100%, there is a steady transformation seen by a wavelength shift from 554 to 498 nm (Figure 4D). Sensitization occurs as follows: RhB and Rh110 are photoexcited from their ground states to high-energy singlet excited states with excited-state oxidation potentials of approximately −1.3 V (RhB*) and −1.5 V (Rh110*).^44,45^ These excited-state potentials lie significantly more negatively than the CB edge of CdSSe/ZnS QDs (−0.8 and −1.04 V vs NHE respectively) and TiO_2_ (−0.5 V vs NHE), enabling thermodynamically favorable electron injection (Gibbs free energy change Δ*G* < 0) from the dye excited states into the QDs or TiO_2_ layer.

The valence band edges of CdSSe/ZnS (1.2/2.5 V vs NHE) and TiO_2_ (2.7 V vs NHE) lie well below the dye oxidation potentials, suppressing back electron transfer. Overall, photoexcitation of the organic dyes elevates the system to a higher Gibbs free energy state, rendering the cascade of electron-transfer steps dye → CdSSe/ZnS or TiO_2_ energetically downhill and facilitating efficient photosensitization and charge separation.

The study presented above was carried out using an equivalent mass of material, which is both economically relevant and the most practical approach for evaluating real-world photocatalysts. However, to assess the intrinsic photocatalytic efficiency of the materials themselves, comparisons can also be made under conditions where the total available surface area is kept constant. Comparing SP and QD, the mass concentration was kept constant; therefore, due to the differences in sizes, the QDs have a larger overall surface area, which is preferential for photocatalysis. Considering the average diameter of the QD and SP, 6 nm and 1.2 μm, respectively, an estimated total surface area in a 1 mL solution at 1.2 mg/mL was found to be 0.18 m^2^ and 0.9 mm^2^ for QD and SP, respectively. This calculates to approximately 195 times larger surface area for the QD solution compared to SP. Therefore, it is not surprising that under UV, QD was able to fully degrade all rhodamine derivatives in only 150 min, where SP still had 12% dye remaining after 300 min. To compare the photocatalytic efficiencies of SP and QD, the sample concentrations were adjusted to maintain a constant available surface area. As a result, the QD dispersion was diluted to a concentration of 6 μg/mL so the total available surface area for both samples was kept consistent at approximately 0.9 mm^2^. Figure S11 demonstrates that when the available surface area is comparable, SPs outperform QDs, retaining only 12% of the dye compared to 60% for QDs. SPs offer several photophysical advantages over QDs, which allow them to operate as more efficient photocatalysts than their subcomponents. Their micron-scale size induces Mie resonances, which enhance scattering and increase photon dwell times. Additionally, the collective behavior of QDs within the SP structure promotes more efficient charge separation, reducing recombination relative to dispersed QDs. Together, these factors contribute to the superior photocatalytic efficiency of SPs compared to QDs.

#### 3.3.2 White Light Irradiation

The photocatalysis measurements were also performed under white light illumination. As with the UV experiments, the concentration of the photocatalysts was kept constant between samples at 1.2 mg/mL and the concentration of RhB was 20 μM. Figure 6 displays the absorbance spectra of the extracted RhB dyes when mixed independently with the photocatalysts and irradiated under white light. All samples showed rhodamine degradation via *N*-deethylation, noted by the hypsochromic shift to 498 nm associated with the transformation to Rh110.

As with the UV light study, the percentage of dye degradation was plotted as a function of time (Figure 7). Interestingly, the rate of degradation showed a different trend with QD compared to SP, SP/TiO_2_, and TiO_2_. For the QD sample, the RhB absorbance drops approximately 40% over a 90 min period (Figure 7A) with the peak absorption wavelength residing around 550 nm, indicating the dominance of RhB (Figure 7D). Between 90 and 150 min of light irradiation, there is a sharp decrease in the absorbance down to 8% of the initial value. This drop happens concurrently with a blue shift to a peak absorbance wavelength of 500 nm (Figure 7D), indicating that Rh110 is now the dominant species. The hypsochromic shift occurs at the same time the QD solution becomes noticeably cloudy, with QDs precipitating out of the solution, as shown in Figure S12. We believe this is due to the QD first breaking down the ligands surrounding the nanomaterial, which then allows a higher concentration of RhB to adsorb onto the surface, leading to a greater rate of degradation. The ζ-potential of the QDs was measured after photocatalysis and was found to be −20.7 mV (Figure S13), compared to −31.5 mV before white light irradiation. A ζ-potential less than −30 mV or higher than 30 mV typically indicates a stable solution.^48^ Therefore, the increase in ζ-potential suggests the solution is becoming less stable, which agrees with the observed precipitation.

Although TiO_2_ has a large band gap and is unable to absorb wavelengths above 380 nm, the TiO_2_ NPs were still able to degrade RhB under white light through a self-sensitization route. Due to the highly negative zeta potential of the TiO_2_ NPs (−62 mV), the positively charged RhB molecules will adsorb onto the surface. Again, the CB of the TiO_2_ is more positive than the LUMO of the rhodamine dyes; therefore, electrons can inject into the TiO_2_ CB, allowing TiO_2_ to create ROS even with visible light.^42,49–51^ In the presence of TiO_2_, Rh110 does not degrade at the same rate as RhB. This is due to the spectral overlap of the dye absorption with the emission of the white light lamp (Figure 5). For white light, the RhB absorption spectra have a greater overlap with the spectrum of the white light lamp compared to Rh110, meaning fewer Rh110 molecules will be in the excited state, leading to a lower rate of electron transfer to the photocatalysts compared to RhB.

Similar to UV irradiation for SP/TiO_2_, the dominating mechanism resulting in dye degradation is through self-sensitization of the dye, demonstrated by the increase in absorbance at 498 nm up to 150% from its initial value. However, for SP/TiO_2_ under visible light irradiation, not only can electron transfer occur directly into the TiO_2_ layer from RhB/Rh110 (shown by the red arrow in Figure 5B), but photoexcited electrons from CdSSe/ZnS QDs can inject into the CB of the TiO_2_ layer (≈−0.5 V vs NHE), which serves as a terminal electron acceptor and promotes spatial charge separation.

However, the CB of ZnS (−1.04 V vs NHE) is more negative than the CB of the CdSSe core (−0.8 V vs NHE), meaning electrons cannot readily transfer as it is not thermodynamically favorable (Figure 5B). If the ZnS shell is thin enough (1–3 monolayers), then electrons can tunnel through the ZnS layer, subsequently transferring to the more positive CB of the TiO_2_.^9,10,21^ The holes in the VB are more strongly confined in the CdSSe core and therefore less likely to tunnel through, promoting efficient charge separation, reducing recombination, and potentially increasing photocatalytic efficiency.

The photocatalytic efficiency for SP and QD was again compared by adjusting the QD concentration so that the total surface area of the QD and SP was approximately 0.9 mm^2^. Under white light, the QD showed little RhB degradation, with approximately 90% of the dye still remaining after 300 min (Figure S14). As with UV irradiation, the SPs were able to degrade the dye much more efficiently compared to isolated QDs. This is a key result highlighting the benefits of using SPs as photocatalysts.

### 3.4 Kinetic Rates

The performance of the photocatalysts was evaluated by measuring the kinetics of the RhB molecule. This can be found using the pseudo-first-order kinetic equation, ln­(*C*_0_/*C*) = *kt*, where *k* is the degradation rate and *C*_0_ and *C* is the initial and concentration at time *t*, respectively.^52^ As the samples were left to equilibrate with RhB for 30 min in the dark, the initial concentration, *C*_0_ was defined at *t* = 0 so the kinetic rate reflects only the photocatalytic degradation and not the dark catalysis. Figure S15 shows the plots of ln­(*C*_0_/*C*) as a function of *t*, where the gradient is the kinetic rate for each photocatalyst under UV and white light irradiation. The concentration was found by monitoring the absorbance at 554 nm, which studied only RhB degradation. The results are shown in Table 1 where *R* ^2^ is the goodness of fit.

**Table 1:** Kinetic Rates of RhB in the Presence of Photocatalysts: TiO_2_, QD, SP and SP/TiO_2_ under UV and White Light Irrespectively

	UV light				white light			
	K_1_ (min^–1^)	R^2^_1_	K_2_ (min^–1^)	R^2^_2_	K_1_ (min^–1^)	R^2^_1_	K_2_ (min^–1^)	R^2^_2_
TiO_2_	0.042	0.98	-	-	0.012	0.94	-	-
QD	0.005	1	0.040	0.98	0.003	0.99	0.063	0.87
SP	0.018	0.99	-	-	0.010	0.99	-	-
SP/TiO_2_	0.032	0.98	-	-	0.030	0.98	-	-

Under UV irradiation, the QD shows two distinct rates, both follows the pseudo-first-order kinetics. The first rate is very slow at 0.005 min^–1^ but after 30 min the rate increases 8-fold to 0.04 min^–1^. After 30 min, the QD solution also becomes visibly cloudy because QDs begin to precipitate (Figure S12). This suggests that during the first 30 min under UV irradiation, the QD is only degrading a small concentration of RhB as the ROS are mostly degrading the TGA ligands bound to the QD surface. This leads to colloidal instability and precipitation of the QDs. However, once the surface ligands have been destroyed, the RhB has more available surface area to adsorb to the QD, resulting in an increase in the kinetic rate. This was not observed for the other photocatalysts, as they all showed one rate for the entirety of the experiment. As expected, TiO_2_ exhibited the fastest degradation rate under UV irradiation (0.042 min^–1^), only slightly outperforming the QD’s second kinetic rate of 0.040 min^–1^. SP/TiO_2_ had a faster rate of 0.032 min^–1^ compared to SP’s rate of 0.018 min^–1^. This is due to a number of factors. First, the more negative zeta potential of SP/TiO_2_ (−21 mV) compared to SP (−12 mV) attracts more cationic RhB to the surface. Second, at an equivalent concentration, SP/TiO_2_ exhibits greater UV absorbance (Figure 5A), which corresponds to a higher rate of excited carrier generation relative to SP. It must also be noted that the same mass concentration was used for these results; therefore, the total number of active sites differs between SP/TiO_2_ and SP.

Under white light, the QD again has two kinetic rates. For the QD, the degradation rate is 0.003 min^–1^ up to 120 min, after which the rate accelerates to 0.063 min^–1^. As with UV light, the change in rate is attributed to the degradation of the organic ligands surrounding the QDs. The second kinetic rate (0.063 min^–1^) is faster under white light than UV (0.04 min^–1^), which could be due to two reasons. First, the irradiance of the white light is almost 2-fold greater than the UV lamp, and second, the UV photons are more likely to be scattered by the QDs and products, therefore leading to a higher absorption in the visible. Under white light, the TiO_2_ NPs show a nonlinear response when fitted with pseudo-first-order kinetics eq (Figure S15), which results in a poor fit with an *R* ^2^ of 0.94. Due to the *N*-deethylation of RhB being the primary mechanism of dye degradation for TiO_2_ under white light illumination, the kinetic rate reflects the degradation of RhB, Rh110, and the intermediate rhodamine derivatives, leading to a nonlinear response.^53–55^

For white light, *k* was found to be 0.030 min^–1^, 0.010 min^–1^, and 0.003 min^–1^ for SP/TiO_2_, SP, and QD (before precipitation), respectively. As with UV irradiation, SP/TiO_2_ outperforms SP under white light by 1.26-fold. This can be attributed to several factors, first, an increase in adsorption of RhB to the surface due to the more negative zeta potential. Therefore, a larger rate of dye molecules will be degraded or transformed to Rh110. Second, as previously discussed, under visible light irradiation, photoexcited electrons from QDs can inject into the TiO_2_ layer, facilitating greater charge separation compared to SP. In addition, the absorption of SP/TiO_2_ has a larger overlap with the emission of the white light lamp compared to SP at the same concentration (Figure 5A), resulting in a greater rate of excited charge carriers.

### 3.5 Reusability

A key requirement that should be considered when engineering a photocatalyst is the reusability of the materials. The reusability was measured by illuminating the sample under both UV and white light and measuring the absorption of the dye, RhB at 554 nm in 30 min intervals over a 3 h period; the photocatalyst was then purified from the dye material via centrifugation. The photocatalyst was again mixed with a fresh solution of RhB and irradiated. Figure 8 shows the recyclability tests, with each photocatalyst undergoing 3 cycles.

**Figure 8:**
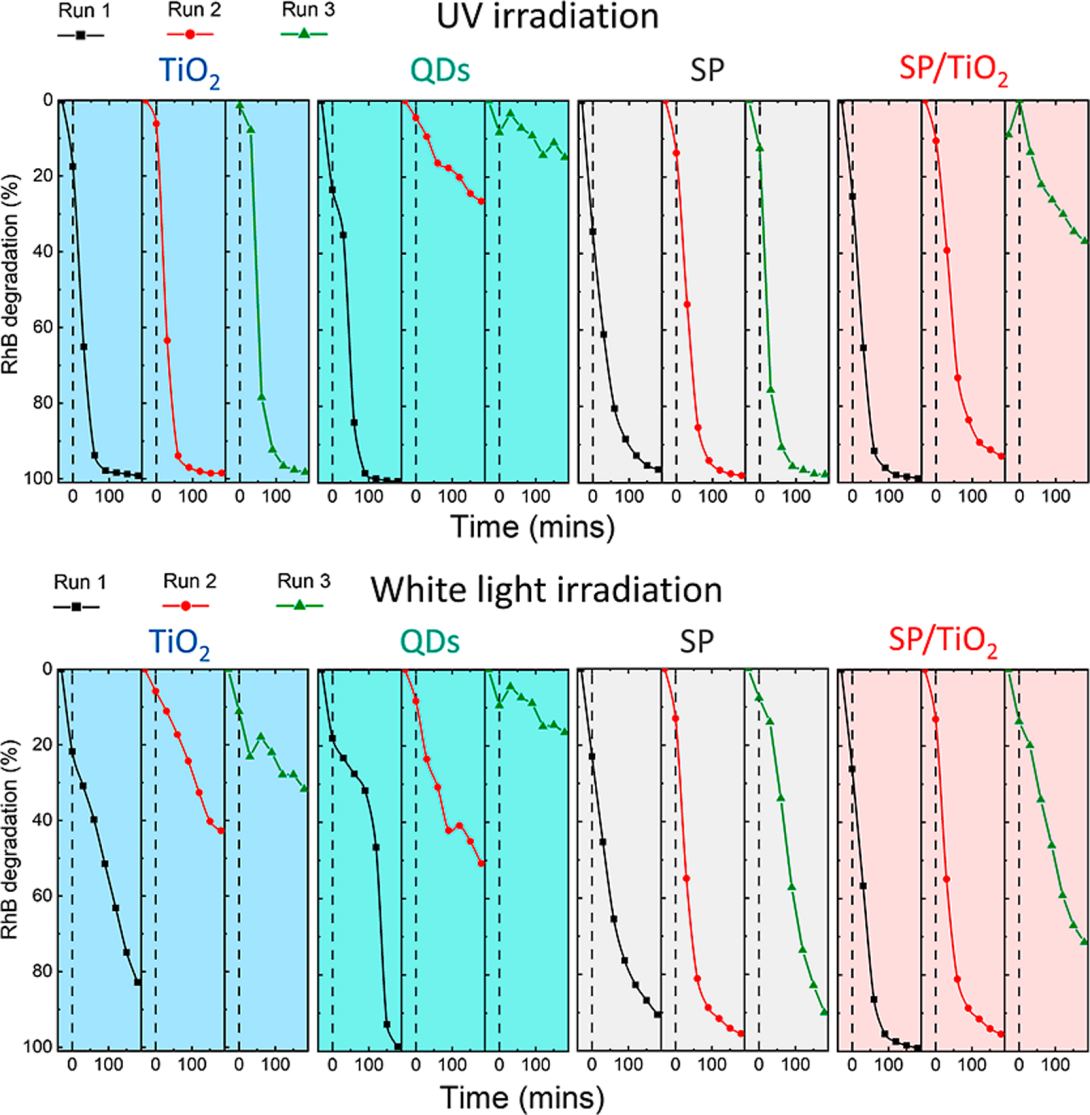
Reusability studies of photocatalysts under UV and white light irradiation. The reusability was measured by monitoring the absorption of RhB at 554 nm over a 180 min period. After which the photocatalyst was precipitated, washed, and resuspended in a fresh 20 μM RhB solution, where the experiment was then repeated again. This was replicated for 3 cycles.

Under UV light, the TiO_2_ showed good reusability with RhB being degraded over a 180 min period for each cycle. However, this was not the case under white light. As discussed earlier, due to the large band gap of TiO_2_, the formation of ROS under white light can only occur due to the self-sensitization of RhB, which results in the formation of Rh110. For TiO_2_, this process is less efficient for each run with the percentage of RhB being degraded reaching only 30% by the third run compared to 82% on the first. The ζ-potentials of the TiO_2_ NPs were measured before and after the 3 irradiation cycles. The ζ-potential had increased from −62 to −16 mV, meaning less RhB will adsorb to the TiO_2_ surface, which would result in a lower photocatalytic efficiency (Figure S16). As with the QDs after photocatalysis, the increase of the ζ-potential, increasing to −16 mV, indicates the TiO_2_ solution is not as stable, which can result in the aggregation of the nanomaterials, potentially reducing the photocatalytic efficiency.

Under both UV and white light, the QD sample showed very little reusability. As discussed earlier, under both excitation wavelengths, the QD suffered instability as the surface ligands were degraded. This was shown by an increase in the ζ-potential (Figure S13), and visually, the solution became cloudy and the QDs began to precipitate, indicating irreversible aggregation (Figure S12) whereby the QDs lost ligands and possibly underwent photocorrosion. This process has been well documented for chalcogenide QDs such as CdS.^11,22,23,56^ Photogenerated holes migrate to the surface and can cause hole-driven oxidation reactions of surface sulfide ions. The accumulation of excess holes is the main cause of the low photostability of QDs, thereby massively affecting their reusability. In stark contrast, SPs showed excellent reusability under both light sources. This could be due to several reasons. First, the SP architecture offers greater mechanical strength, which could mitigate photocorrosion effects.^12,14^ Second, owing to the micrometer-scale dimensions of the SP, the excitation light is attenuated by a combination of absorption by the QDs and Mie scattering within the dense, high-refractive-index structure. As a result, QDs near the surface are preferentially photoexcited, while those embedded deeper within the structure experience reduced excitation and, consequently, less photocorrosion. This is a key result indicating the benefit of colloidal QD suprastructures over individual QDs. SEM images of the SPs before and after photocatalysis (Figure S17) show that although after 3 cycles under UV irradiation the SPs have become less spherical, they still retain their supraparticle form.

SP/TiO_2_ showed good reusability for runs 1 and 2 under both light sources; however, run 3 was not as effective, with only 70% of RhB being degraded compared to approximately 95% for runs 1 and 2 for white light and reaching only 38% under UV light. SEM images of SP/TiO_2_ were taken before and after reusability tests (Figure S18), where the photocatalyst was reused for 3 cycles under white light. The images show that after 3 cycles, SP/TiO_2_ begins to physically fragment into smaller clusters. Elemental analysis was obtained for this sample (Figure S19), and showed spatial correlation between the cadmium and titanium, indicating that the titania shell was simply not just breaking away from the SP core, but the whole structure as a whole was beginning to fragment.

## 4 Conclusion

This work demonstrates the use of quantum dot supraparticles as an alternative to traditional TiO_2_ nanoparticles and colloidal quantum dots. QD-based structures outperform TiO_2_ under white light, monitored by the degradation of RhB, demonstrating the potential of these materials as visible light photocatalysts. We also demonstrate that when the concentrations of SPs and QDs are of comparable total surface area, SPs outperform the QDs by degrading the RhB much faster. In addition, the formation of SPs from individual QDs dramatically increases the photostability of the QD material, with SPs showing little degradation after three photocatalytic run cycles. This is a key result to potentially overcome photocorrosion effects in colloidal quantum dots, which impacts their use as photocatalysts. The addition of the titania shell also changes how RhB is degraded, with the *N*-deethylation forming Rh110 being the preferred route for SP/TiO_2_.

## Supplementary Material



## Data Availability

Data set can be found at: 10.15129/b18be2e4-53da-4b33-bd19-b74ff0c08557

## Notes

Size distribution of SP and SP/TiO_2_, EDX maps of SP, SP/SiO_2_, and SP/TiO_2_, zeta potential results of SP, SP/SiO_2_, SP/TiO_2_, QD-TGA, and TiO_2_ before and after white light illumination, control measurements of RhB degradation under dark conditions with and without SP, photocatalyst results for QD and SP concentrated to comparable surface area results under UV and white light irradiation, images of QDs before and after both UV and white light illumination, plots of kinetic rates, and SEM images and EDX maps of SP before and after UV irradiation (PDF)

## Abbreviations

QD: quantum dot
SP: supraparticle.
