# Supplementary material for: Quantum Dot Supraparticle Photocatalysts for Photodegradation of Rhodamine B
Source: ACS Omega. 2026 Apr 10;11(15):23125–37. doi: 10.1021/acsomega.5c13184 (PMC13103759; doi:10.1021/acsomega.5c13184)
Supplement: Supplementary file 1 [file ao5c13184_si_001.pdf]

Supplementary Information

Quantum Dot Supraparticle Photocatalysts for Photodegradation of Rhodamine B

Charlotte J. Eling, Nicolas Laurand*

Institute of Photonics, Department of Physics, SUPA, University of Strathclyde, Glasgow, G1 1RD, UK

*charlotte.eling@strath.ac.uk

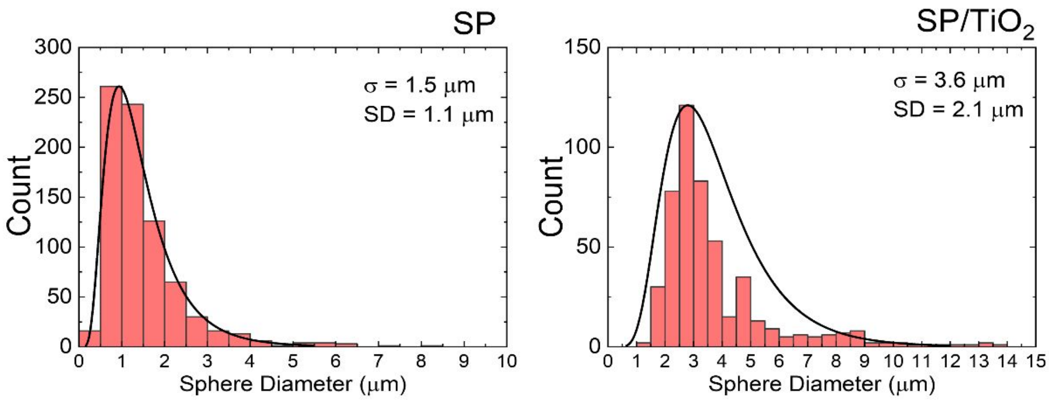

Figure S1: Size distribution of SP and SP/TiO_2. The histogram was fitted with a lognormal curve.

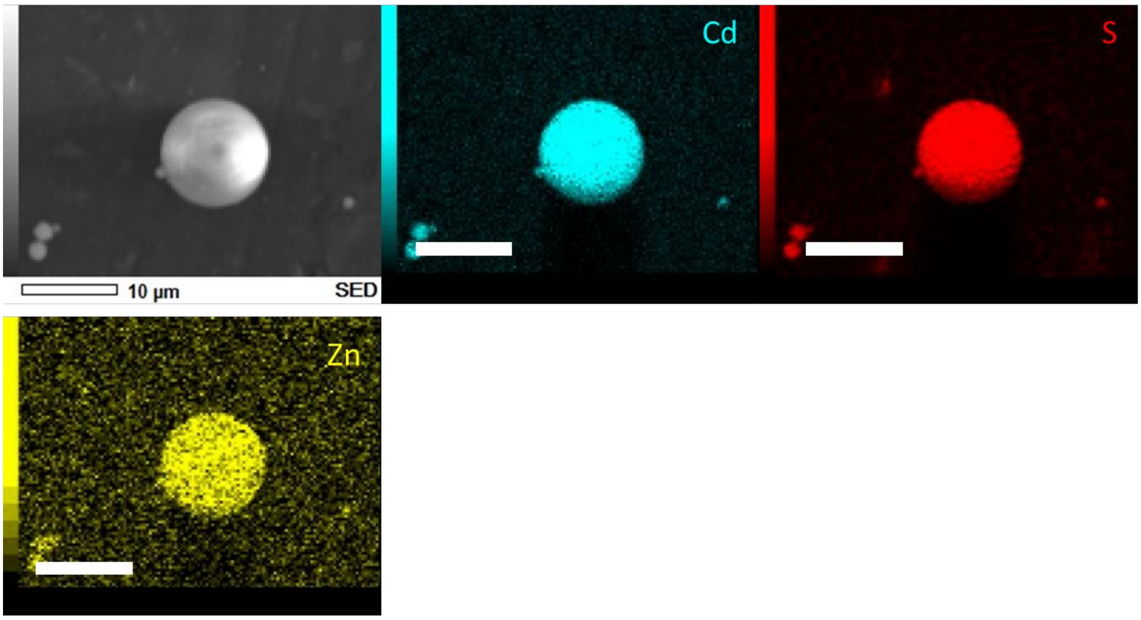

Figure S2: EDX maps of SPs with Cd, S and Zn present. Scale bar is 10 μm.

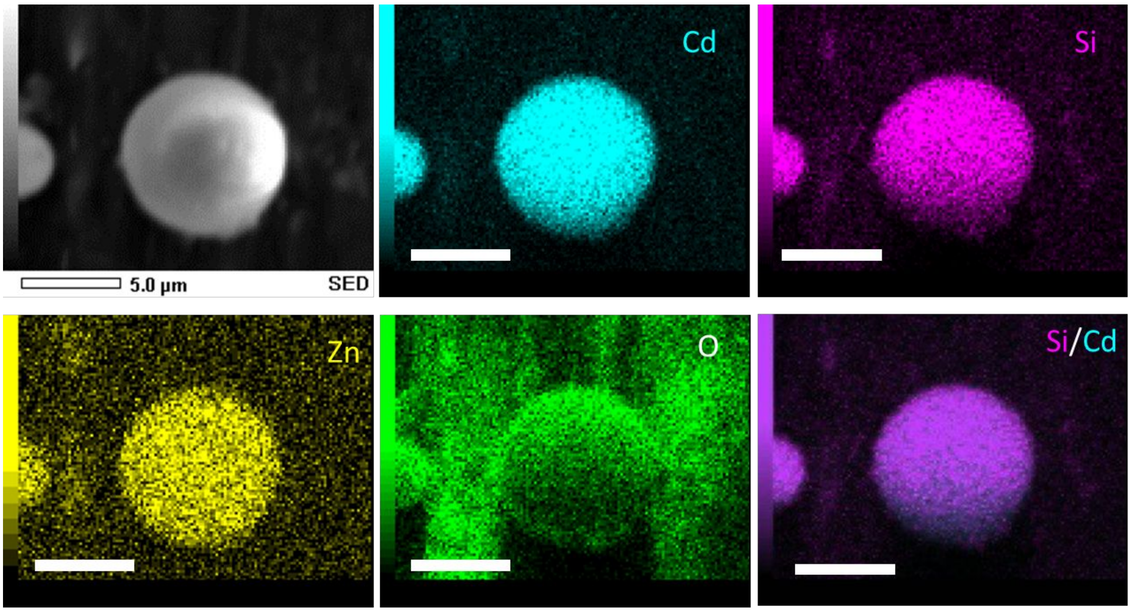

Figure S3: EDX maps of silica coated SP with Cd, Si, Zn and O present. Overlay image of Cd and Si maps show SiO_2 coating of SP. Scale bar is 5 µm.

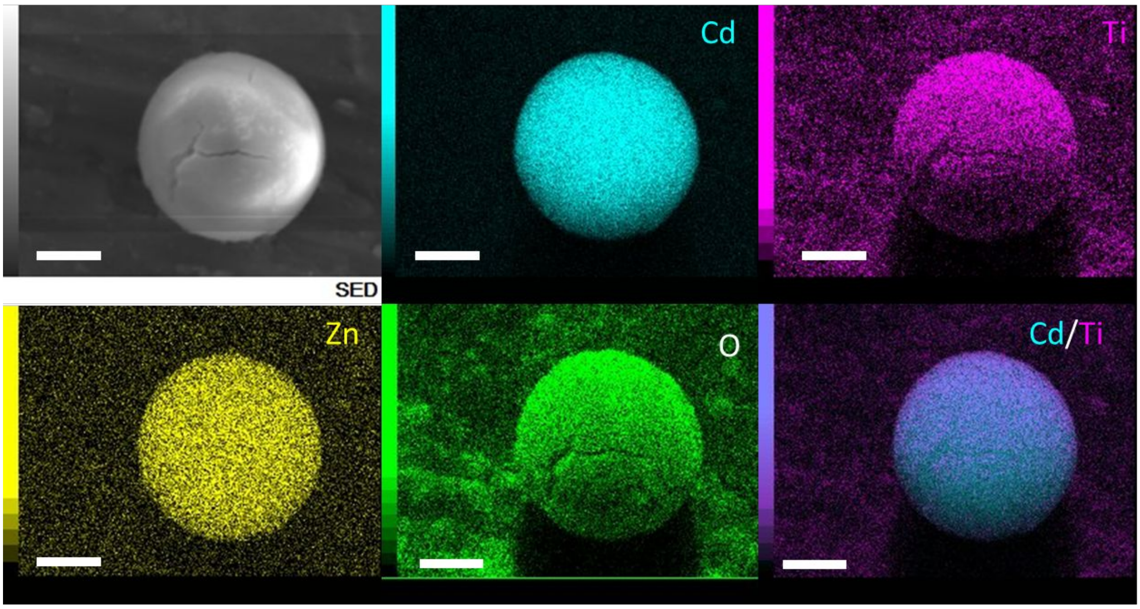

Figure S4: EDX maps of SP/TiO_2, with elemental maps of Cd, Ti, Zn and O. Overlap of Cd and Ti is evidence of the TiO_2 coating on the SPs.

Zeta potential results

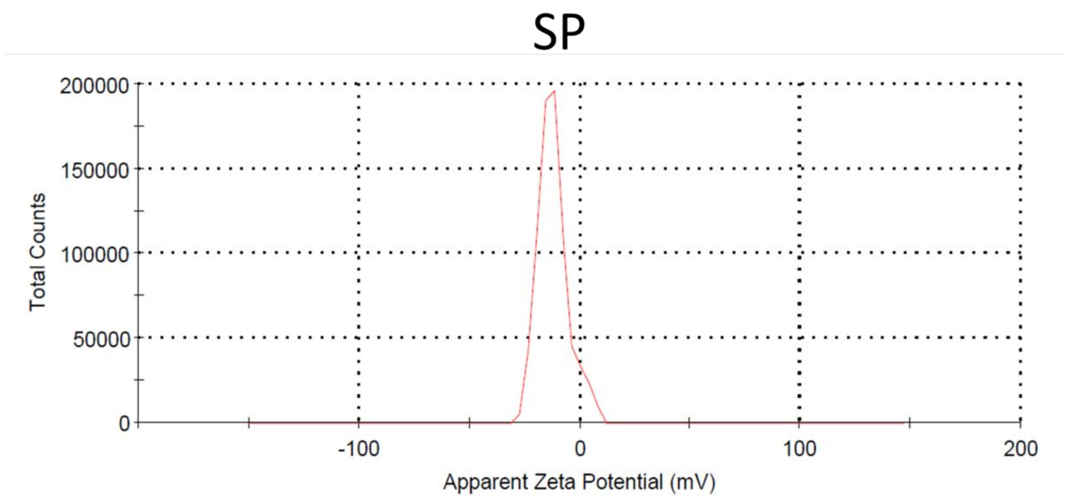

Figure S5: Zeta potential plot of SP found to be -12.2 mV ± 6.9 mV.

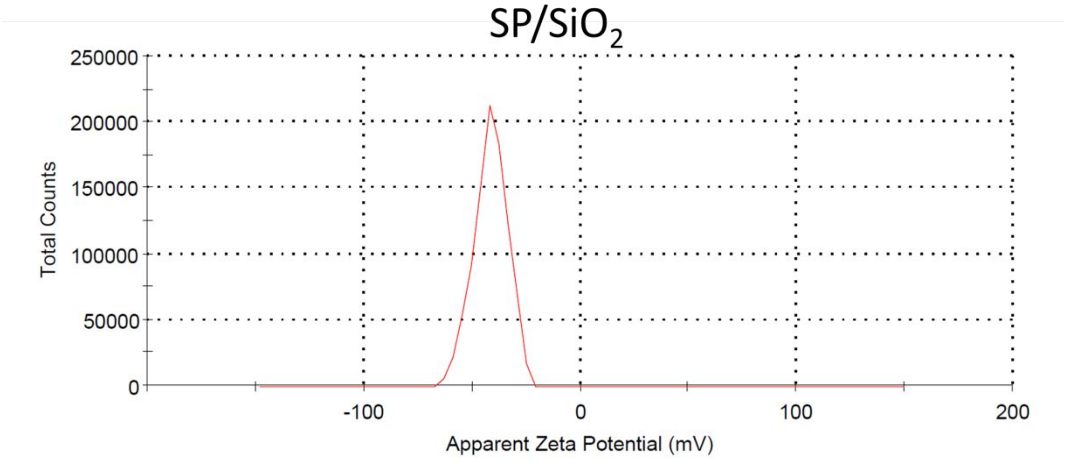

Figure S6: Zeta potential plot of SP/TiO_2 found to be -40.3 mV ± 7.7 mV.

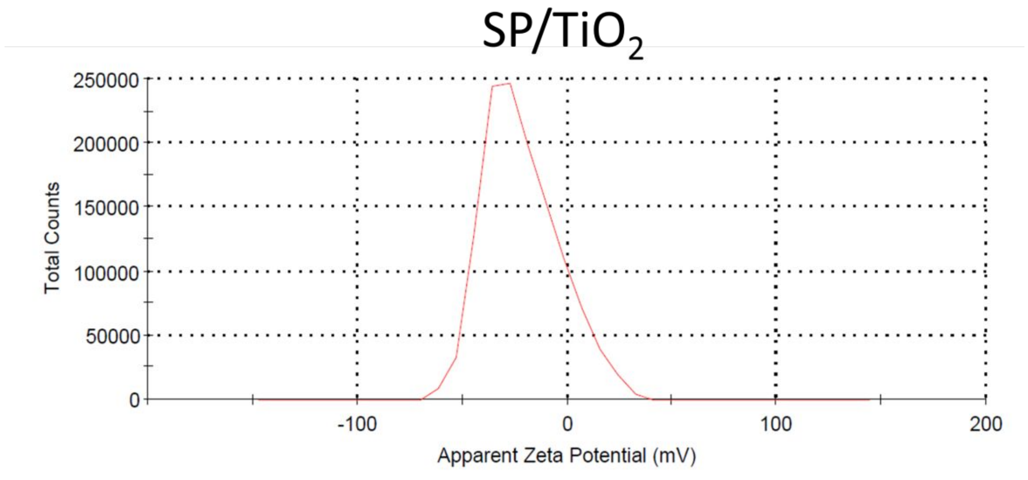

Figure S7: Zeta potential plot of SP/TiO_2 found to be -21.6 mV ± 17.9 mV.

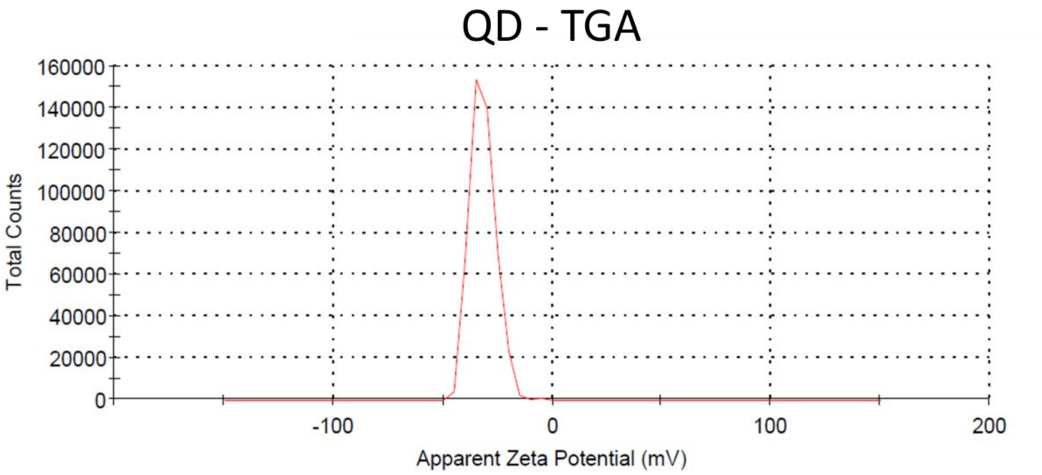

Figure S8: Zeta potential plot of QD found to be -31.5 mV ± 5.7 mV.

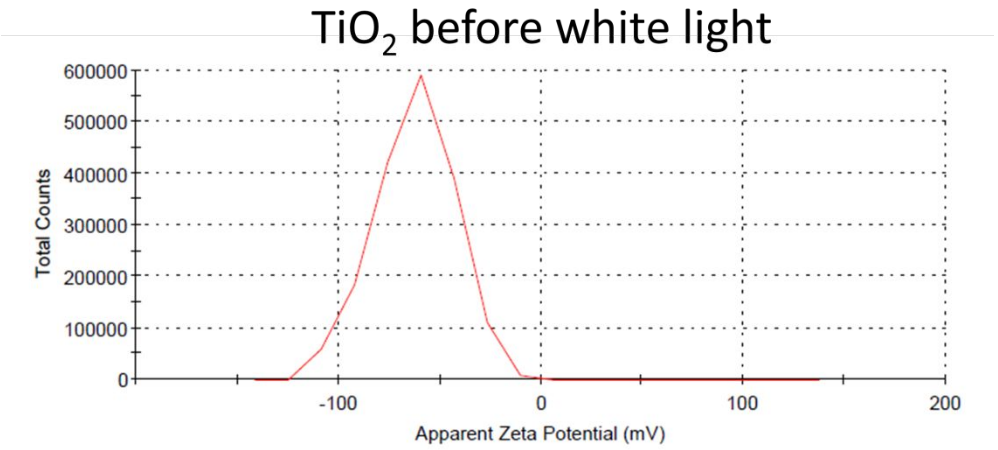

Figure S9: Zeta potential plot of TiO_2 before irradiation, found to be -62.3 mV ± 19.7 mV.

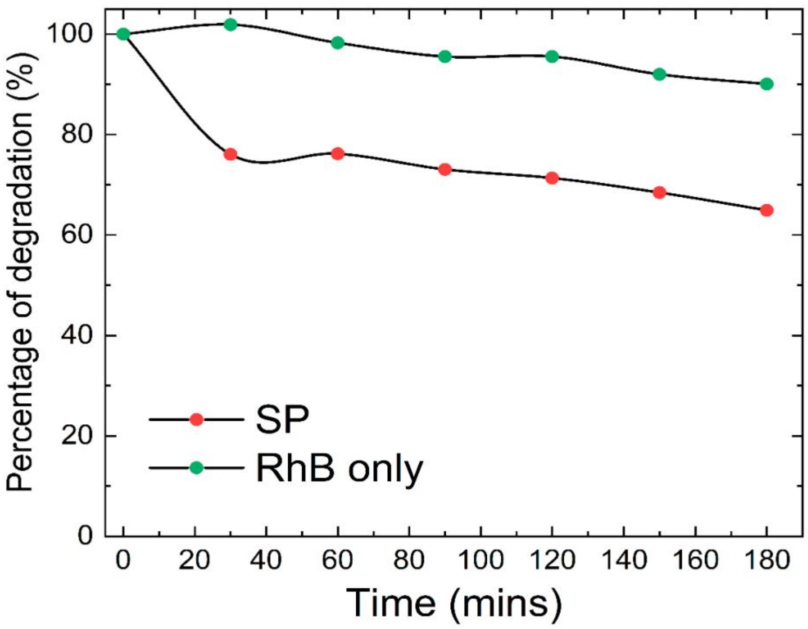

Figure S10: Percentage of RhB degradation with solution of 1.2 mg/mL SP mixed with 20 μM RhB and 20 μM RhB only solution. The solutions were stirred at room temperature and kept in the dark, wrapped in aluminium foil. The absorbance was measured every 30 min over a 3-hour period. The SPs were separated from RhB via centrifugation.

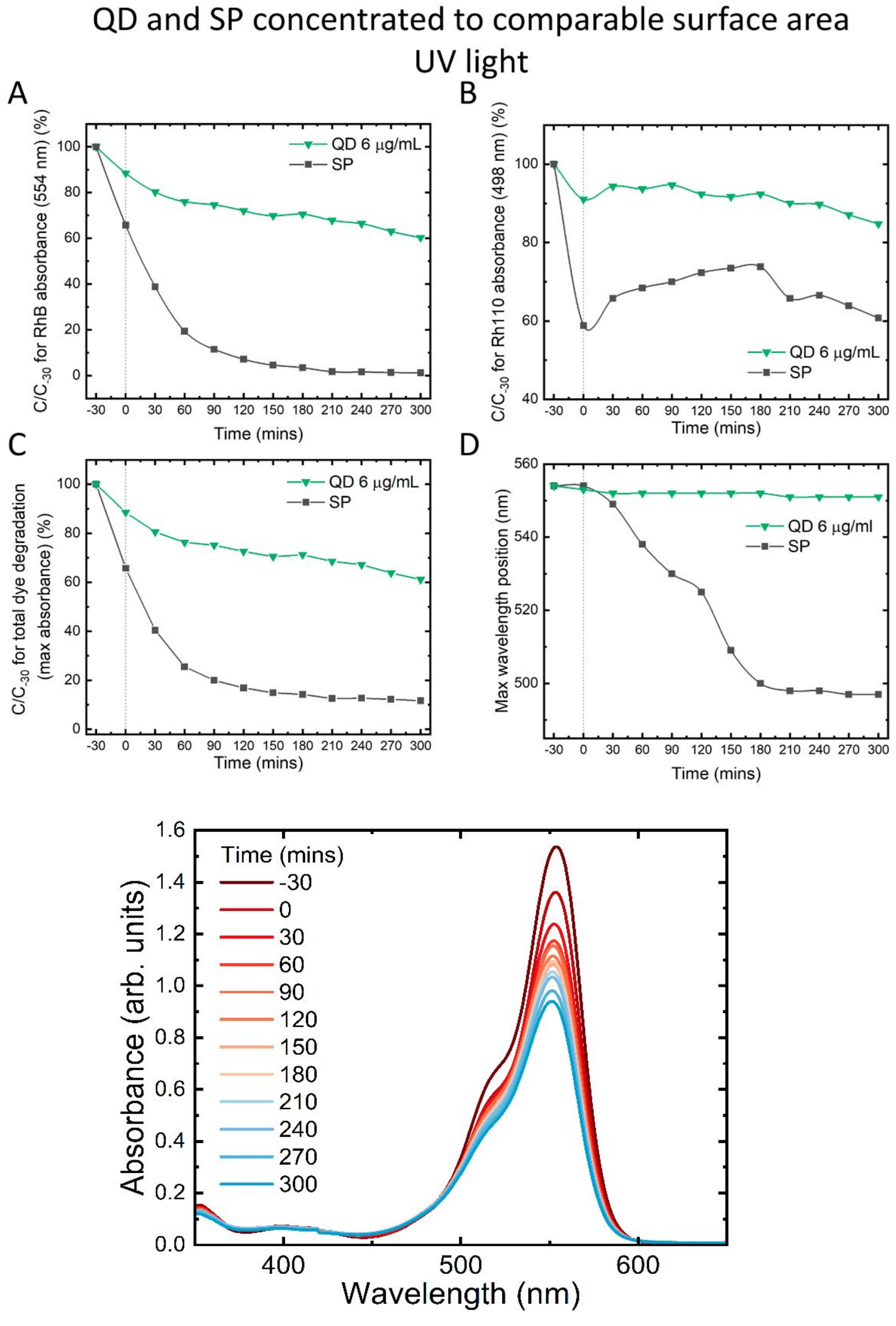

Figure S11: Percentage of photocatalytic degradation of RhB under UV light as a function of time with photocatalysts: QD and SP at a total surface area concentration of 0.9 mm^2. (A) is the absorbance monitored at 554 nm which is the characteristic wavelength of RhB (B) absorbance at 498 nm, characteristic wavelength of Rh110 (C) maximum absorbance,

demonstrating total dye degradation (D) maximum absorbance wavelength, representing the transformation of RhB to Rh110

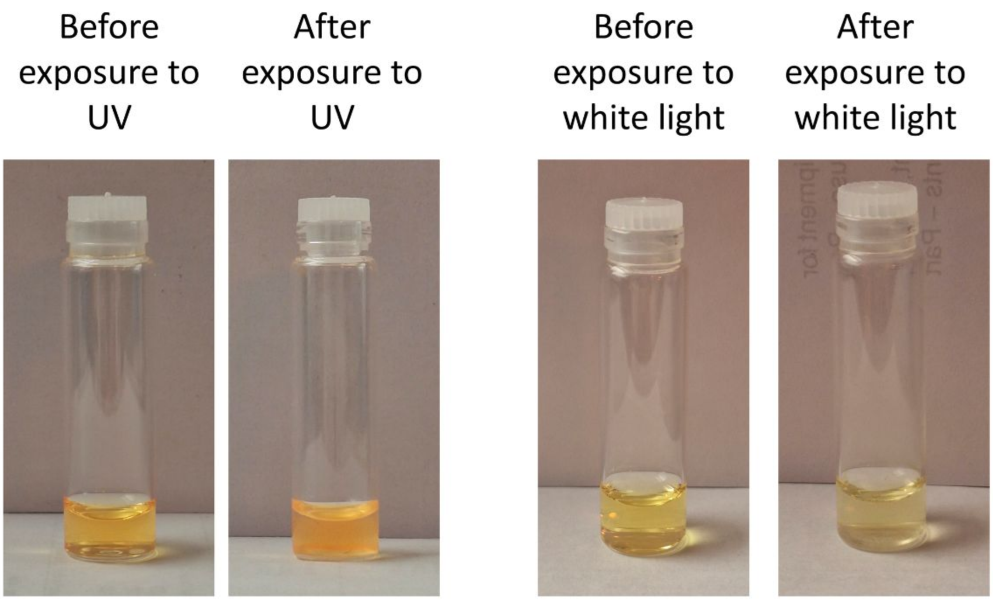

Figure S12: Image of QDs before and after 2 hours exposure to UV and white light respectively. After 2 hours, the solutions have become noticeably cloudy and the QDs begin to precipitate. This is evidence of photodegradation of the QDs, impacting its reusability as a photocatalysts under both white light and UV irradiation

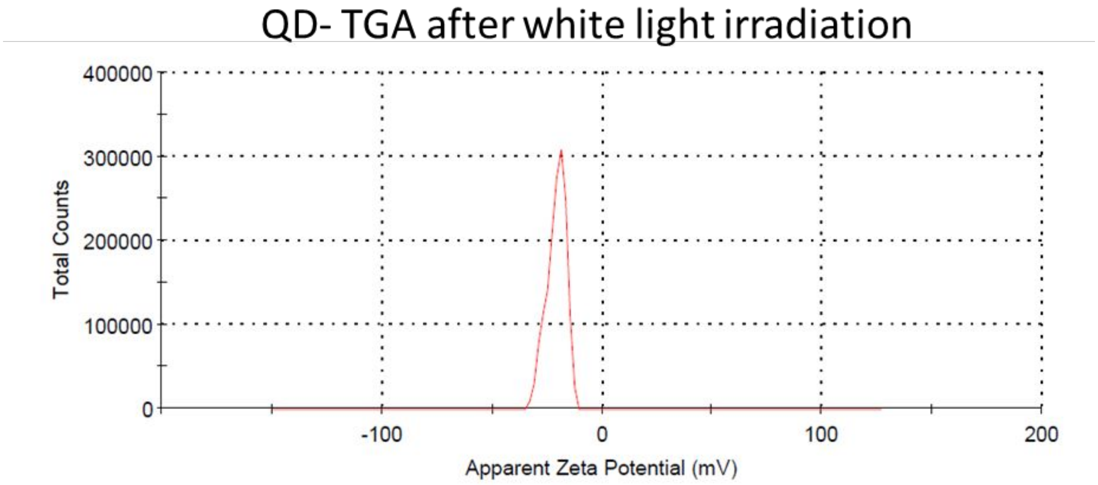

Figure S13: Zeta potential plot of QD-TGA after white light irradiation.

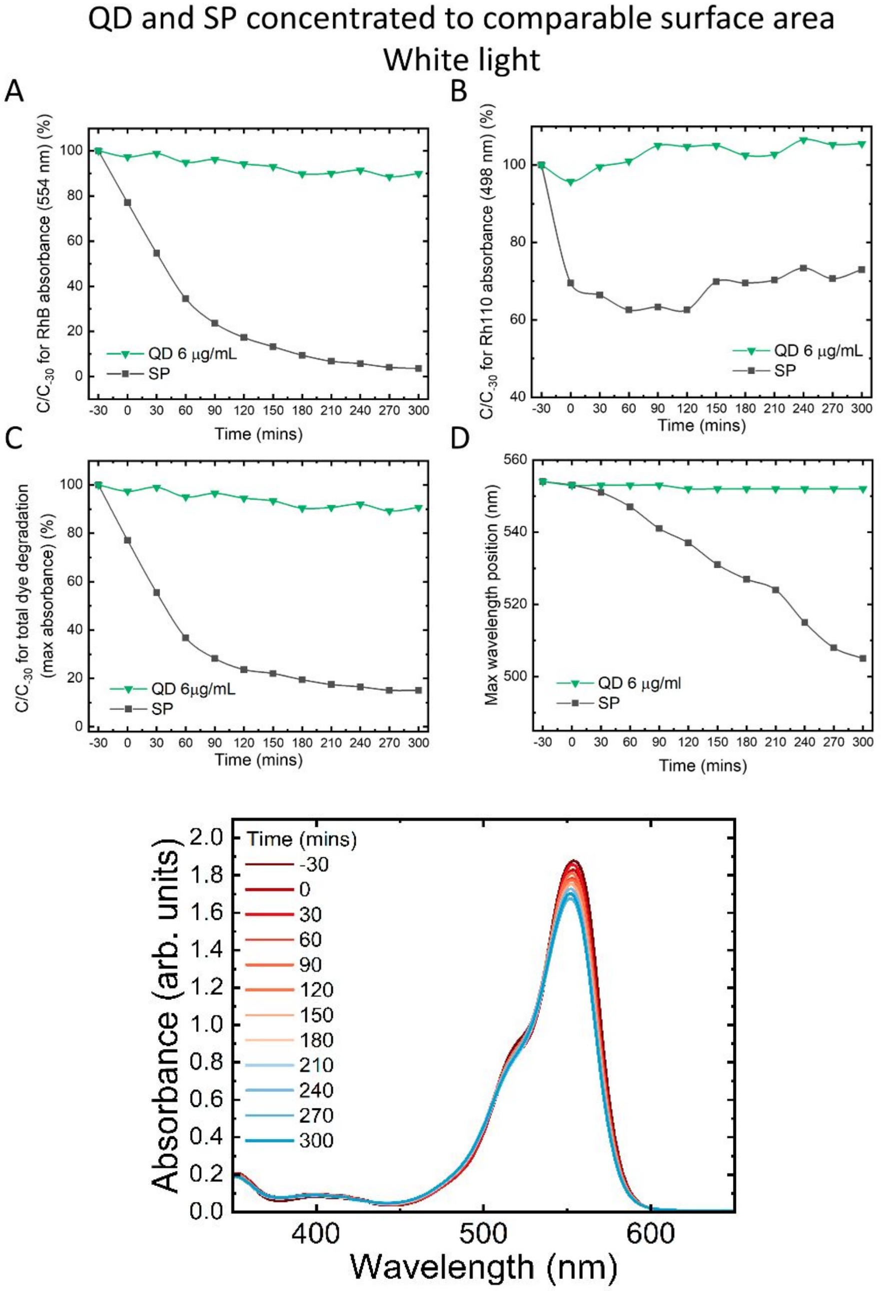

Figure S14: Percentage of photocatalytic degradation of RhB under white light as a function of time with photocatalysts: QD and SP at a total surface area concentration of 0.9 mm^2. (A) is the absorbance monitored at 554 nm which is the characteristic wavelength of RhB (B) absorbance at 498 nm, characteristic wavelength of Rh110 (C) maximum absorbance, demonstrating total dye degradation (D) maximum absorbance wavelength, representing the transformation of RhB to Rh110.

Kinetic equations

The kinetic rate was calculated using the pseudo first order kinetic rate, $\ln(C_0/C) = kt$, where k is the kinetic rate, and C and C_0 is the concentration at time =t and t=0 respectively. The figures show $\ln(C_0/C)$ as a function of time for each photocatalyst under both UV and white light

irradiation. The gradient represents the kinetic rate. The fit was only calculated during times where the RhB was degrading. The point at which the absorbance of RhB (at 554 nm) reached zero (Figure S5) is shown by the grey box. Once all the RhB had been degraded, the data no longer exhibited a linear trend, therefore only the data corresponding to RhB degradation is fitted.

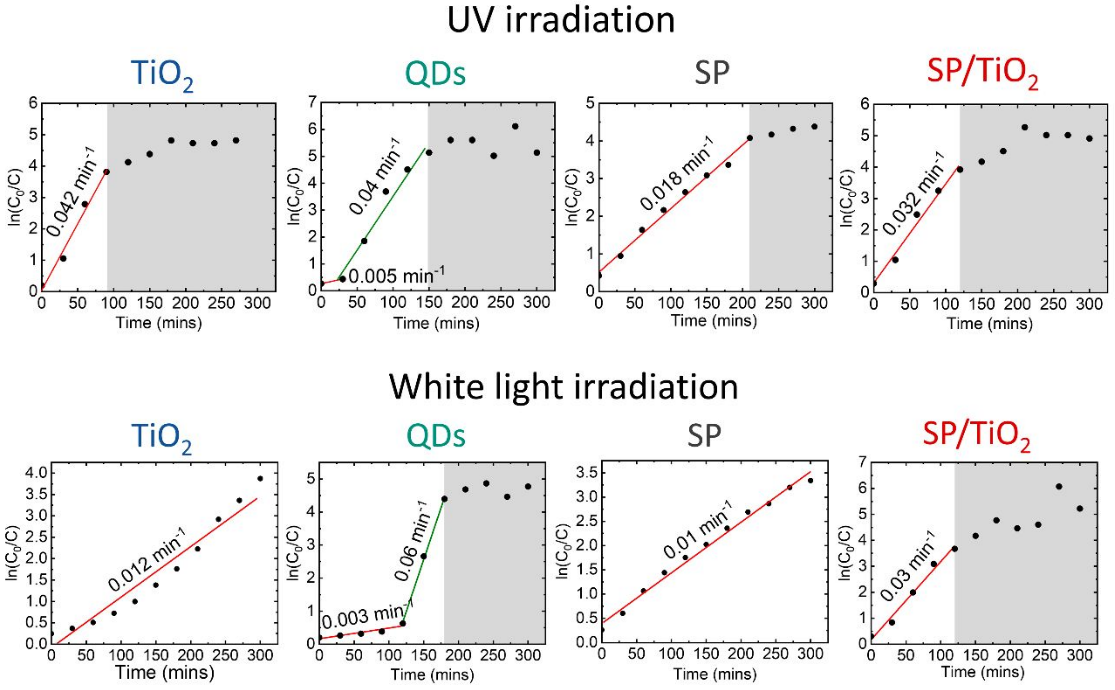

Figure S15: Degradation rates of photocatalysts under either UV or white light irradiation. The grey segment indicates where the dye had been fully degraded therefore no longer followed the linear trend.

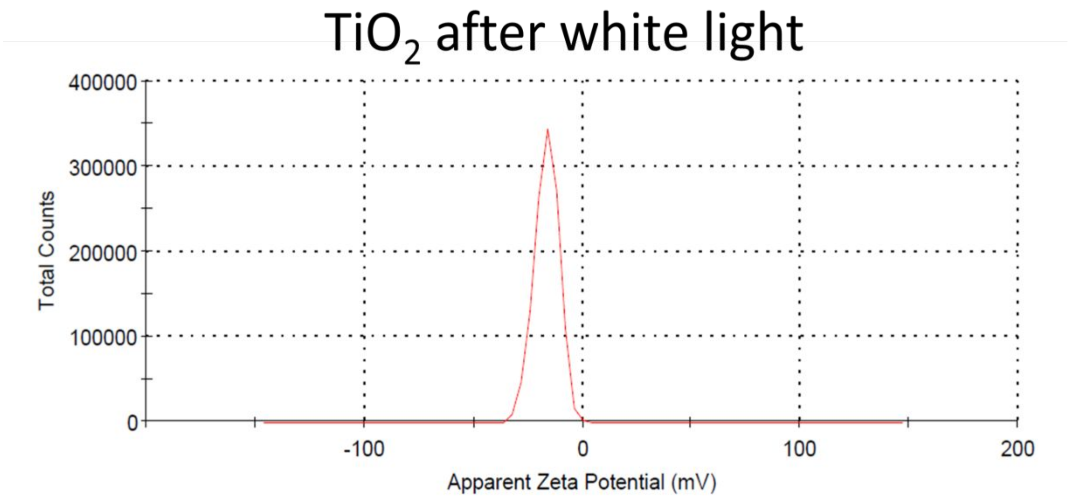

Figure S16: Zeta potential plot of TiO_2 after 3 rounds of photocatalysis measurements under white light illumination. The Zeta potential was found to be -16.6 mV ± 4.8 mV.

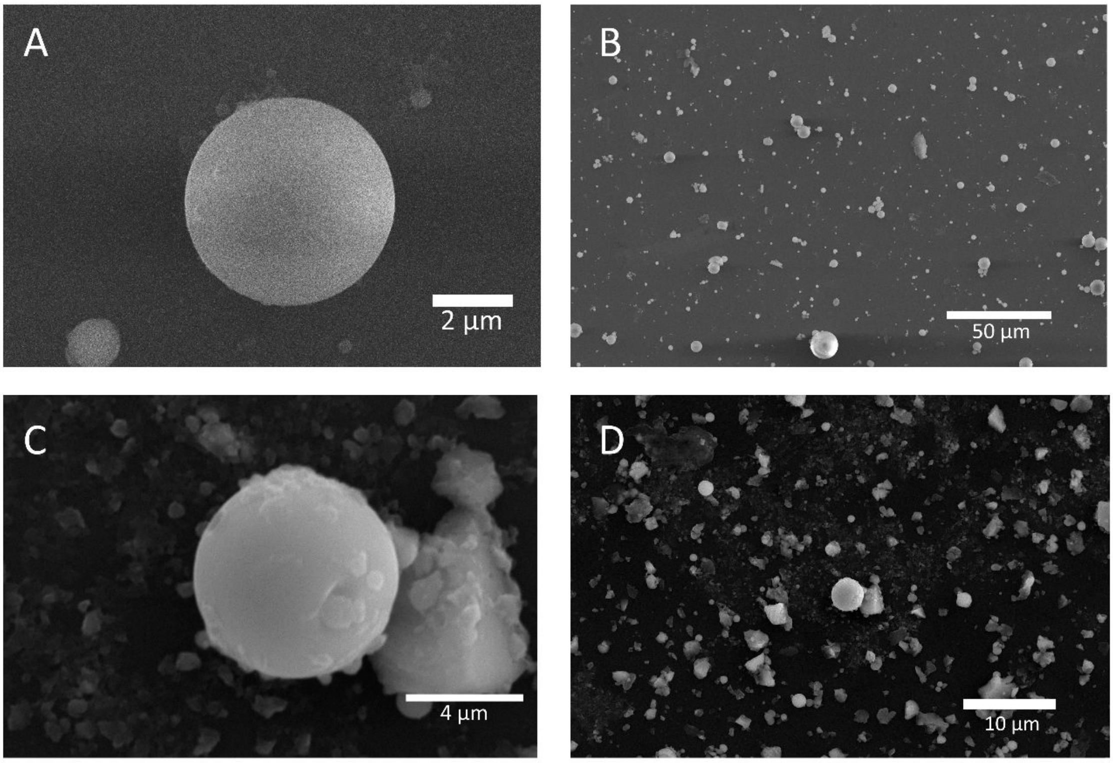

Figure S17: A, B, SEM images of SP before white light irradiation. C, D SEM images of SP after three cycles of white light irradiation.

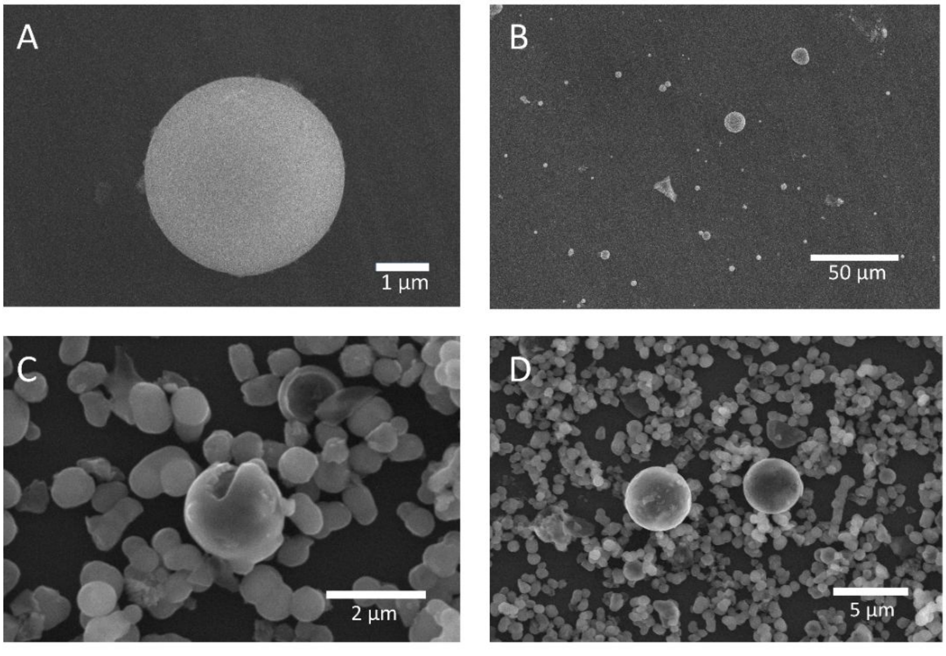

Figure S18: A, B, SEM images of SP/TiO_2 before white light irradiation. C, D SEM images of SP after three cycles of white light irradiation.

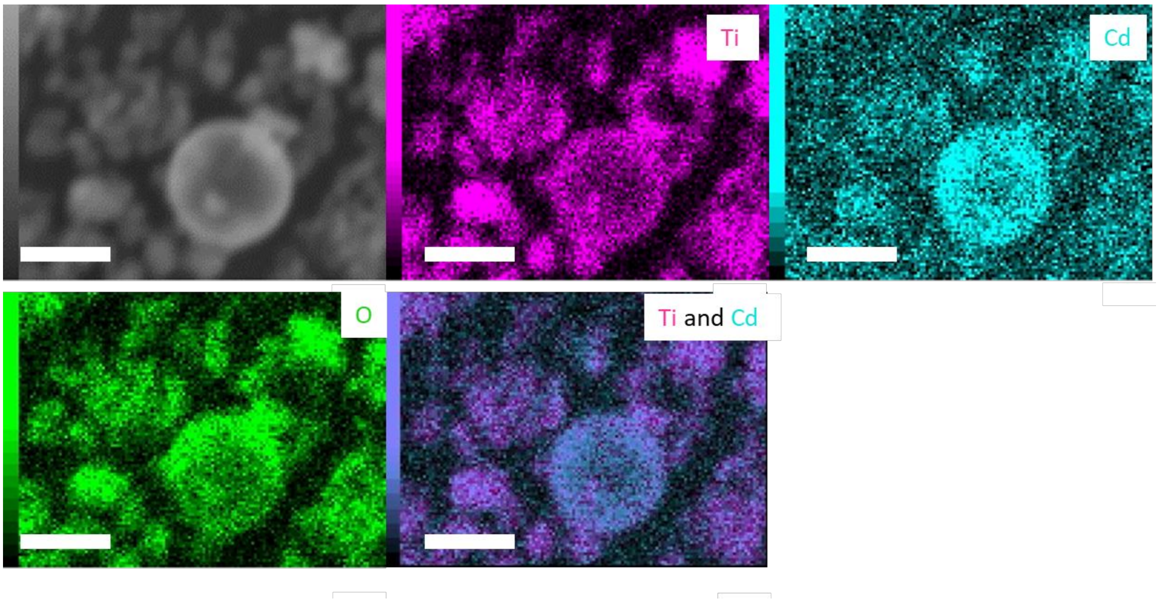

Figure S19: EDX maps of SP/TiO_2 after 3 cycles of white light irradiation. Elemental maps of Ti, Cd, O and Ti/Cd overlay. Scale bar is 3 μm.
